# Isoliensinine confers neuroprotection and alleviates LPS-induced neuroinflammation in microglia by regulating the MAPK/NF-κB signaling

**DOI:** 10.3389/fphar.2025.1675865

**Published:** 2025-12-10

**Authors:** Mengqin Yuan, Jinda Hu, Lichen Gao, Wei Long, Sirui Wang, Xinyue Tan, Jinyue Hu

**Affiliations:** 1 Department of Pharmacy, School of Pharmaceutical Science, Phase I Clinical Trial Centre, The Affiliated Changsha Central Hospital, Hengyang Medical School, University of South China, Hengyang, Hunan, China; 2 Hunan Provincial Key Laboratory of Tumor Microenvironment Responsive Drug Research, Hengyang, Hunan, China; 3 School of Life Science, Hunan University of Science and Technology, Xiangtan, China; 4 Medical Research Center, The Affiliated Changsha Central Hospital, Hengyang Medical School, University of South China, Changsha, China

**Keywords:** Alzheimer’s disease, isoliensinine, LPS, MAPK/NF-κB, neuroinflammation, neuroprotection

## Abstract

**Background:**

The increasing aged population poses issues in the management of age-related disorders, notably Alzheimer’s disease (AD), which significantly affects the health and quality of life of seniors. Neuroinflammation is a significant factor in Alzheimer’s disease pathogenesis. Isoliensinine (ISO), a bisbenzylisoquinoline alkaloid derived from lotus seed embryos, exhibits antioxidant and anti-inflammatory effects. Nonetheless, its function in neuroinflammation has yet to be investigated.

**Methods:**

We examined the impact of ISO on LPS-induced neuroinflammation in BV2 microglial cells by using biological tests. Western blotting confirmed ISO’s influence on MAPK/NF-κB signaling pathways. In addition, oxidative stress markers and JC-1 staining were employed to assess the impact of ISO on LPS-induced oxidative stress and mitochondrial dysfunction in BV2 cells.

**Results:**

ISO markedly diminished LPS-induced neuroinflammation in BV2 cells through the modulation of the MAPK/NF-κB pathway. Conditioned media derived from ISO-treated BV2 cells enhanced the vitality of HT-22 cells. ISO also alleviated oxidative stress and mitochondrial dysfunction.

**Conclusion:**

Our findings indicate that ISO mitigates neuroinflammation by inhibiting MAPK/NF-κB signaling and provides neuroprotection by diminishing oxidative stress and mitochondrial impairment. These effects collectively enhance its neuroprotective capacity, indicating that ISO may represent a potential candidate for further investigation in AD.

## Graphical Abstract

**Figure:**
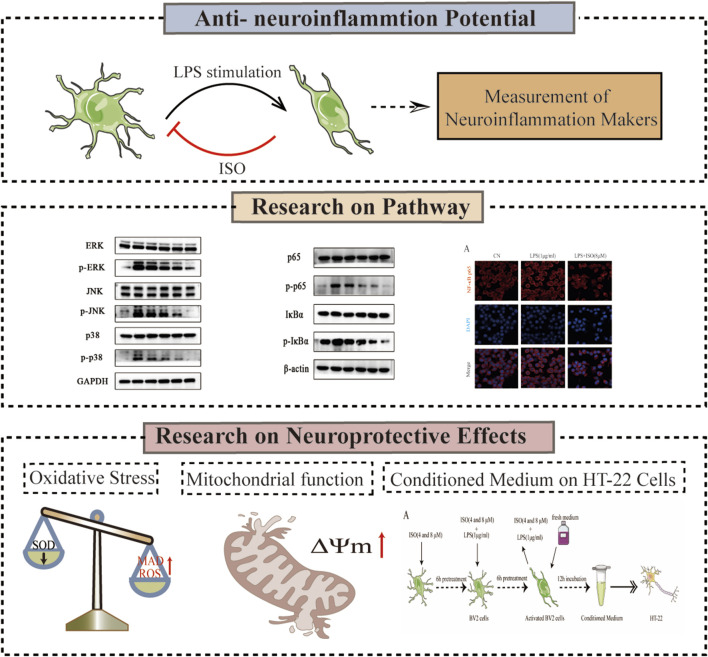
The experimental flowchart outlines the combination of biological methods, such as Western Blotting, oxidative stress marker assessment, and JC-1 mitochondrial membrane potential assay, to investigate the neuroprotective mechanism of ISO.

## 1 Introduction

The accelerating global demographic shift toward aging populations presents formidable challenges in addressing age-related diseases. Alzheimer’s disease (AD), the most common neurodegenerative dementia globally, severely affects both physical health and quality of life in elderly adults. Current epidemiological estimates indicate that roughly 55 million adults worldwide suffer from dementia, with AD comprising 35 million cases (60%–70% of total dementia prevalence) (Pereira et al., 2024). Beyond its devastating clinical manifestations, AD imposes multidimensional burdens, including profound familial caregiving strain and substantial socioeconomic consequences. These compelling factors underscore the imperative for advancing both early diagnostic modalities and novel therapeutic interventions for AD, representing one of the most pressing biomedical and public health challenges of our era (Chen et al., 2024).

Isoliensinine (ISO) is a bisbenzylisoquinoline alkaloid primarily isolated from the lotus seeds’ core (Shu et al., 2015). This plant has been widely used in traditional Chinese medicine for its calming, antipyretic, and cardioprotective effects. Recent phytochemical studies have identified ISO as one of the major bioactive constituents responsible for these therapeutic benefits (Cheng et al., 2021). Furthermore, comprehensive pharmacological investigations have revealed that ISO has promising applications in antioxidant (Long et al., 2024), anti-tumor (Chen et al., 2025), and cardiovascular protection (Yao et al., 2024). For instance, ISO inhibits the proliferation and migration of gastric cancer cells by targeting the TGFBR1 signaling pathway (Hu et al., 2024) and induces apoptosis and cell cycle arrest in various cancer cell lines, including breast (Zhang et al., 2015) and lung cancer cells (Chen et al., 2025), demonstrating promising anticancer potential. Nevertheless, in recent years, studies have also revealed the neuroprotective effects of ISO, which can reduce apoptosis and cell death in PC12 cells induced by Aβ25-35 (Meng et al., 2022). Moreover, in animal models of AD (Li et al., 2025), ISO significantly alleviates neurotoxicity induced by β-amyloid and improves cognitive function. Therefore, these findings highlight the importance of further elucidating the molecular mechanisms underlying ISO’s neuroprotective effects.

Recent studies have emphasized the significant role of neuroinflammatory processes in the development of AD. Specifically, amyloid beta (Aβ) deposition and tau protein fibrillary tangles are critical catalysts that initiate the innate immune response, thereby leading to the release of inflammatory mediators (Chen and Yu, 2023). These mediators not only accelerate the pathological progression of AD but also worsen its severity. Furthermore, the continuous release of inflammatory factors results in neuronal dysfunction and death, which further exacerbates cognitive decline. Collectively, these findings underscore the pivotal role of neuroinflammation as a primary pathogenic mechanism in AD (Heneka et al., 2025). Moreover, the inflammatory response disrupts the clearance of Aβ and tau proteins, thus creating a vicious cycle that perpetuates the disease process (de Oliveira et al., 2021).

Microglia, the intrinsic immune cells of the central nervous system (CNS), are important for preserving homeostasis and responding to pathological insults (Navarro et al., 2018). In particular, under inflammatory conditions, such as those induced by lipopolysaccharide (LPS), microglial activation triggers a cascade of neuroinflammatory responses that are closely linked to the progression of neurodegenerative diseases (Leng and Edison, 2021). LPS, a potent agonist of Toll-like receptor 4 (TLR4), activates key intracellular pathways, including the mitogen-activated protein kinase (MAPK) and nuclear factor kappa B (NF-κB) pathways (Ju et al., 2021). Consequently, this activation results in the production of pro-inflammatory cytokines and reactive oxygen species (ROS), which contribute to neuronal damage and dysfunction.

The MAPK and NF-κB signaling pathways engage in intricate crosstalk that critically contributes to the neuroinflammatory processes observed in AD. Within this interplay, the MAPK pathway—comprising P38 MAPK, c-Jun N-terminal kinase (JNK), and extracellular signal-regulated kinase (ERK)—plays a pivotal role in microglial activation. Specifically, Aβ accumulation activates the TLR4 receptor, which subsequently triggers the p38 MAPK pathway. This activation, in turn, enhances the release of pro-inflammatory cytokines such as tumor necrosis factor-alpha (TNF-α), interleukin-1 beta (IL-1β), and interleukin-6 (IL-6), thereby exacerbating neuroinflammation (He et al., 2020). Notably, hyperactivation of JNK not only drives inflammatory cytokine production but also induces tau hyperphosphorylation, leading to the formation of neurofibrillary tangles and ultimately resulting in neuronal dysfunction and death (Ploia et al., 2011). Interestingly, the ERK1/2 pathway exerts dual effects in AD (Wei et al., 2024): although its activation supports neuronal survival and synaptic plasticity through its neuroprotective role, it simultaneously enhances the transcriptional activity of AP-1, which upregulates pro-inflammatory cytokines and aggravates neuroinflammation.

Similarly, NF-κB activation stimulates microglial activation and drives the release of various pro-inflammatory cytokines and chemokines, thereby amplifying neuroinflammatory responses (Wang et al., 2025). Moreover, NF-κB facilitates Aβ production and tau phosphorylation, further aggravating AD pathogenesis. Critically, the MAPK and NF-κB pathways do not function independently but engage in reciprocal regulation. For example, p38 MAPK activates NF-κB by inducing IκB degradation, while NF-κB reciprocally upregulates MAPK kinase expression (Falcicchia et al., 2020). Collectively, the MAPK/NF-κB pathway integrates Aβ/tau toxicity, oxidative stress, and cytokine networks, serving as a pivotal hub in AD neuroinflammation and representing a promising therapeutic target.

A diverse array of natural compounds have been shown to attenuate neuroinflammation and consequently delay the progression of AD, largely owing to their anti-inflammatory and neuroprotective properties. Among these, curcumin mitigates neuroinflammation by inhibiting the NF-κB and MAPK signaling pathways, thereby downregulating pro-inflammatory cytokine expression and enhancing cognitive function (Azzini et al., 2024). Likewise, resveratrol, a compound with well-established antioxidant and anti-inflammatory activities, modulates the SIRT1/NF-κB signaling axis to reduce neuroinflammatory responses (Cong et al., 2021). In addition, quercetin has demonstrated the ability to suppress activation of the NLRP3 inflammasome and regulate Toll-like receptor (TLR) pathways, exerting both neuroprotective and anti-neuroinflammatory effects *in vitro* and *in vivo* (Bianchini et al., 2025). Collectively, these findings underscore the significant therapeutic potential of natural compounds in the prevention and management of AD. Of particular interest, ISO exhibits both antioxidant and anti-inflammatory activities and has been found to protect neuronal cells against glutamate-induced cytotoxicity (Long et al., 2024). However, the precise molecular mechanisms underlying its neuroprotective effects remain to be elucidated and therefore warrant further in-depth investigation.

In this investigation, the murine microglial BV2 cell line was used as an experimental model to investigate the anti-inflammatory effects of ISO and the underlying signaling mechanisms. Our initial findings demonstrated that ISO can attenuate LPS-induced neuroinflammation. To date, the effects of ISO on the MAPK and NF-κB signaling pathways in the BV2 neuroinflammatory model have not been evaluated. Therefore, we hypothesized that ISO may mitigate LPS-induced inflammation in BV2 cells by downregulating the activation of MAPK and NF-κB pathways. Moreover, to further explore the neuroprotective properties of ISO, we also examined whether ISO could alleviate LPS-induced oxidative stress and mitochondrial dysfunction. Taken together, our results indicate that ISO alleviates LPS-induced neuroinflammation and neurotoxicity, likely via inhibition of the MAPK/NF-κB signaling pathways. This line of evidence lays a foundation for future investigations into the neuroprotective potential of ISO in neuroinflammatory and neurodegenerative diseases.

## 2 Materials and methods

### 2.1 Cell culture

BV2 microglial cells were provided by Abiowell Biotechnology Co., Ltd. (Changsha, China). HT-22 cells were obtained from the Institute of Clinical Pharmacology, Central South University (Changsha, China). All cells used in this study were maintained between passages 3 and 10. Both cell lines were cultured in Dulbecco’s Modified Eagle Medium (DMEM) (Gibco, United States) supplemented with 10% (v/v) heat-inactivated fetal bovine serum (FBS) (Gibco, United States), 100 U/mL penicillin, and 100 μg/mL streptomycin at 37 °C in a humidified incubator containing 5% CO_2_. To prepare the conditioned medium (CM) from BV2 cells, the culture supernatant was collected after the indicated treatments and centrifuged to remove any cellular debris. The resulting supernatant was then mixed with fresh DMEM at a 1:1 ratio to obtain the conditioned medium. This CM was subsequently used to treat HT-22 cells under the same culture conditions.

### 2.2 Experimental reagents

ISO was acquired from Solarbio Bioscience & Technology Co., Ltd. (Shanghai, China). U0126 (cat no. BA 2003) was purchased from APExBIO Technology LLC. (Houston, TX, United States). Both reagents were dissolved in dimethyl sulfoxide (DMSO; MP Biomedicals Asia Pacific Pte Ltd., CA, United States). Lipopolysaccharide (LPS, *Escherichia coli* 055:B5) was purchased from Solarbio Bioscience & Technology Co., Ltd. (Shanghai, China). A stock solution of LPS was prepared by dissolving the powder in nuclease-free water to a final concentration of 1 mg/mL, and aliquots were stored at −20 °C until use. NF-κB p65 antibody (cat. No. 8242; 1:1,000) and Phospho-NF-κB p65 (cat. No. 8242; 1:1,000) were purchased from Cell Signaling Technology (CA, United States). The GAPDH (cat. No. AB0037; 1:10000), β-actin (cat. No. AB0038; 1:10000), p38 MAPK (cat. No. CY5256; 1:1,000) Antibody, p-p38 MAPK (cat. No. BY0106; 1:1,000) Antibody, JNK1/2/3 (cat. No. CY5409; 1:1,000) Antibody, p-JNK1/2/3 (cat. No. CY5541; 1:1,000) Antibody, IκB alpha (cat. No. CY5026; 1:1,000) Antibody, p-IκB alpha (cat. No. CY7246; 1:1,000) Antibody and Goat anti-rabbit IgG (cat. No. AB0101; 1:10000) were obtained from Abways (Shanghai, China). The ERK1/2 (cat. No. 343830; 1:1,000) antibody and p-ERK1/2 (cat. No. 301245; 1:1,000) antibody were purchased from ZEN-BIOSCIENCE (Chengdu, China).

### 2.3 Cell proliferation assay

The effect of ISO on the viability of BV2 microglial cells was assessed using the Cell Counting Kit-8 (Yisheng Biotechnology Co., Ltd., Shanghai, China) according to the manufacturer’s protocol. BV2 cells were seeded into 96-well plates at a density of 5 × 10^3^ cells/well and allowed to adhere overnight in complete DMEM. Cells were then treated with various concentrations of ISO (DMSO, 0, 2, 4, 8, 16, 32, and 64 μM) for 6 h, 12 h, and 24 h, respectively. At the end of each incubation period, 10 μL of CCK-8 reagent was added to each well and incubated at 37 °C for 1–2 h in the dark. The absorbance was measured at 450 nm using a microplate reader called Epoch (BioTek Instruments, Inc., Vermont, United States).

### 2.4 Quantitative real-time polymerase chain reaction

A total of 2 × 10^5^ cells in 2 mL of complete medium were seeded into each well of a 6-well plate and cultured overnight. Then the cells were pretreated with ISO for 2 h, followed by stimulation with LPS (1 μg/mL) for 6 h. Total RNA was extracted using TRIzol™ Reagent (Invitrogen, United States) in accordance with the manufacturer’s protocol. Reverse transcription was performed using the RevertAid First Strand cDNA Synthesis Kit (Thermo Fisher Scientific, United States) to synthesize complementary DNA (cDNA) from 1 μg of total RNA. Quantitative PCR was then carried out using FastStart Essential DNA Green Master (Roche, Switzerland) on The LightCycler® 96 Instrument (Roche, Switzerland). The relative expression levels of IL-6, IL-1β, and TNF-α mRNA were normalized to GAPDH using the 2^−ΔΔCt method. All experiments were performed in triplicate, and primer sequences are provided in Table 1.

**TABLE 1:** Sequences of the primers for qRT-PCR.

Gene	Primer sequences
IL-1β	F: CACTACAGGCTCCGAGATGAACAAC R: TGTCGTTGCTTGGTTCTCCTTGTAC
IL-6	F: CTTCTTGGGACTGATGCTGGTGAC R: TCTGTTGGGAGTGGTATCCTCTGTG
TNF-α	F: CTATGGCCCAGACCCTCACA R: TCTTGACGGCAGAGAGGAGG
GAPDH	F: TGGGCTACACTGAGGACCACT R: GGGAGTGTCTGTTGAAGTCG

### 2.5 Nitric oxide (NO) assay

NO production was quantified using a Nitric Oxide Assay Kit (Beyotime Biotechnology, China), based on the Griess reaction, in accordance with the manufacturer’s instructions. Briefly, cells were seeded at a density of 2 × 10^5^ cells per well in 6-well plates and cultured overnight. The cells were then pre-treated with ISO for 2 h, followed by stimulation with LPS (1 μg/mL) for 24 h. After treatment, the cell culture supernatants were collected and incubated with the Griess reagents provided in the kit. The absorbance of the resulting azo dye was measured at 540 nm using a microplate reader. Finally, the NO concentration was calculated based on a standard curve generated from known concentrations of sodium nitrite.

### 2.6 Measurement of oxidative stress markers

BV2 microglial cells were seeded at a density of 2 × 10^5^ cells per well in 6-well plates and cultured overnight. After different treatments, the cells were collected for the measurement of oxidative stress markers. To evaluate the effects of ISO on oxidative stress in LPS-stimulated BV2 microglial cells, malondialdehyde (MDA) content, superoxide dismutase (SOD) activity, and intracellular reactive oxygen species (ROS) levels were assessed using corresponding commercial kits. The MDA level was determined using a Lipid Peroxidation MDA Assay Kit (Nanjing Jiancheng Bioengineering Institute, China), which is based on the thiobarbituric acid (TBA) reaction, following the manufacturer’s protocol. SOD activity was measured using the Total Superoxide Dismutase Assay Kit (WST-8 method; Beyotime Biotechnology, China). Intracellular ROS levels were quantified using the Reactive Oxygen Species Assay Kit (DCFH-DA; Beyotime Biotechnology, China). All assays were performed strictly according to the manufacturers’ instructions.

### 2.7 Membrane potential (ΔΨm) assay

BV2 microglial cells were seeded at a density of 2 × 10^5^ cells per well in 6-well plates and cultured overnight. The cells were pre-treated with ISO for 12 h, followed by stimulation with LPS (1 μg/mL) for 6 h. After treatment, the mitochondrial membrane potential was measured using the JC-1 Mitochondrial Membrane Potential Assay Kit (Beijing Solarbio Science & Technology Co., Ltd., China), according to the manufacturer’s protocol. After JC-1 staining at 37 °C for 20 min in the dark, cells were washed with JC-1 staining buffer, and fluorescence was detected using a fluorescence microscope (BioTek Instruments, United States). JC-1 monomers and aggregates were measured at excitation/emission wavelengths of 488/530 nm (green) and 525/590 nm (red), respectively. The red-to-green fluorescence intensity ratio was calculated to assess changes in ΔΨm.

### 2.8 Western blot analysis

Cells were seeded in six-well plates at a density of 2 × 10^5^ cells per well and allowed to adhere overnight. After drug treatment, the cells were washed gently with cold PBS to remove residual medium and treatment compounds, and then placed on ice. Each treatment group was lysed for 10 min using RIPA buffer (Beyotime Biotechnology, China) supplemented with PMSF and phosphatase inhibitors. Protein concentrations were determined using the BCA Protein Assay Kit (Epizyme Biotech, Shanghai, China) according to the manufacturer’s instructions. Equal amounts of protein (20–30 μg) were separated by 10% SDS–PAGE and transferred onto polyvinylidene fluoride (PVDF) membranes (Millipore, United States) at 100 V for 90 min. The membranes were then blocked with 5% bovine serum albumin (BSA, Bovigen, China) in TBST (Tris-buffered saline containing 0.1% Tween-20) for 1 h at room temperature to prevent nonspecific binding. After washing with TBST, the PVDF membranes were incubated in direct contact with the designated concentration of primary antibodies (1:500–1:2000, as described in the 2.2 Experimental reagents) at 4 °C overnight. The membranes were then washed three times with TBST (10 min each) and incubated with appropriate HRP-conjugated secondary antibodies for 1 h at room temperature. After three additional washes with TBST (10 min each), the protein bands were visualized using enhanced chemiluminescence (ECL) reagents (Epizyme Biotech, Shanghai, China) and imaged using an Azure Biosystems imaging system (United States). Band intensities were quantified using ImageJ software. All antibody concentrations and incubation conditions were optimized to achieve the best signal-to-noise ratio. Band densities for phospho-proteins were normalized to the band densities of their respective total proteins. The resulting ratios were used for statistical comparison. GAPDH or β-actin was used as a loading control to confirm consistent protein loading across all samples.

### 2.9 Immunofluorescence staining

Immunofluorescence staining was performed to assess NF-κB nuclear translocation using the NF-κB Activation Nuclear Translocation Assay Kit (Beyotime Biotechnology, China, Cat. # SN368) according to the manufacturer’s instructions with modifications. Briefly, BV2 cells were seeded onto 15-mm confocal dishes at a density of 1 × 10^5^ cells per dish and allowed to adhere overnight. Cells were pre-treated with ISO for 2 h, followed by stimulation with LPS for 6 h. After treatments, cells were fixed with 4% paraformaldehyde for 10 min and then washed thrice with PBS. After permeabilization with 0.3% Triton X-100 for 5 min, the cells were washed with PBS, blocked in PBS with 5%BSA at room temperature for 1 h. Subsequently, cells were incubated with the primary antibody against the NF-κB p65 subunit provided in the kit overnight at 4 °C. The next day, after being washed with PBS, cells were incubated with a Cy3-conjugated secondary antibody for 1 h at room temperature in the dark. Nuclei were counterstained with DAPI for 10 min. Finally, an appropriate amount of anti-fade mounting medium was applied directly to the cells in the confocal dish. Fluorescence images were captured using a Leica Stellaris 8 confocal microscope (Leica Microsystems, Germany).

### 2.10 Statistical analysis

All experimental data are presented as mean ± standard deviation (SD) from at least three independent experiments. Statistical analyses were performed using GraphPad Prism 9.0 software. Comparisons between two groups were conducted using an unpaired Student’s t-test, while comparisons among multiple groups were performed using one-way analysis of variance (ANOVA) followed by Tukey’s *post hoc* test. A p-value of less than 0.05 was considered statistically significant.

## 3 Results

### 3.1 ISO attenuates LPS-Induced cytotoxicity in BV2 cells

**FIGURE 1:**
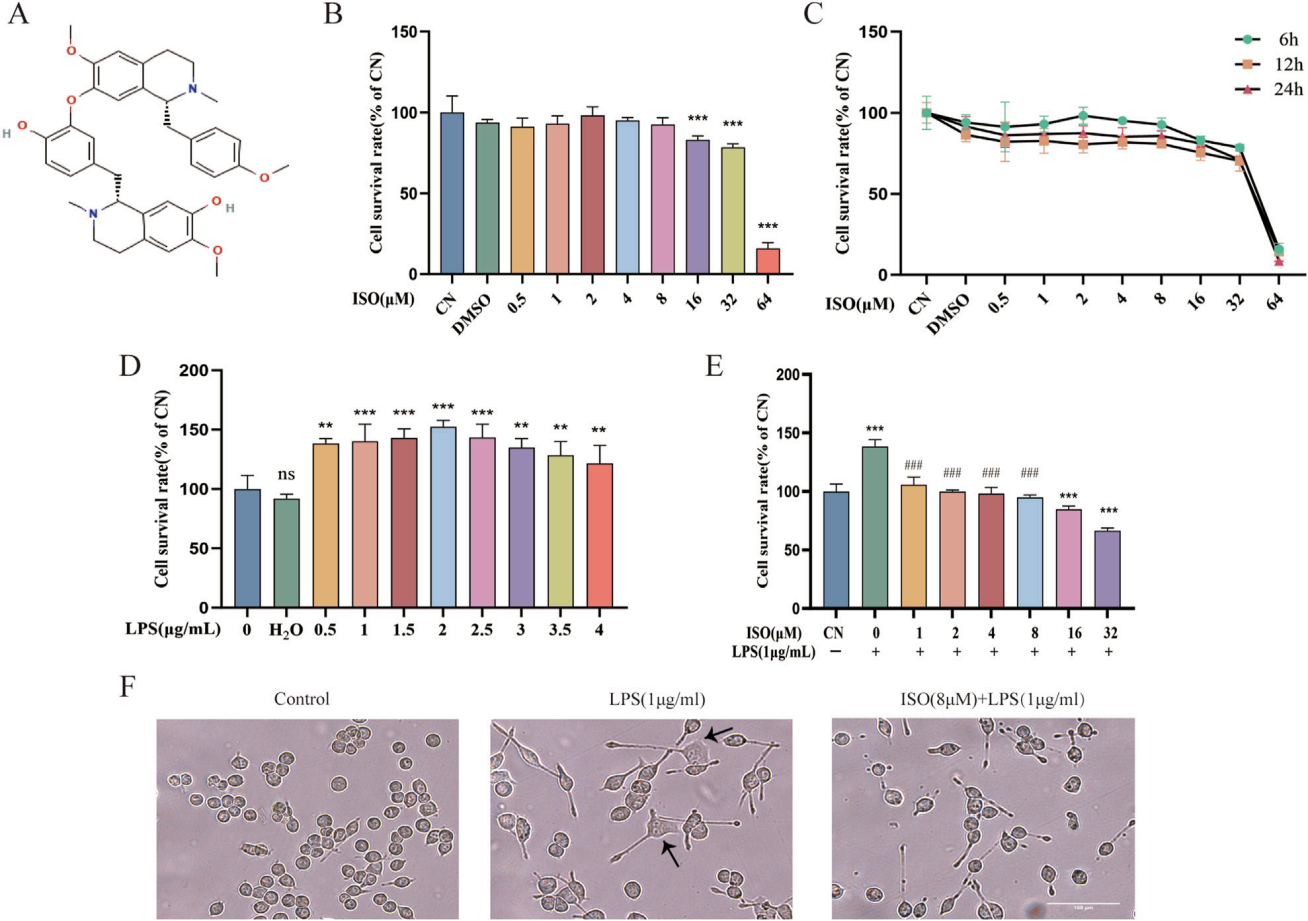
Effects of ISO on the viability and morphology of BV2 cells with or without LPS stimulation. **(A)** Chemical structure of ISO. **(B,C)** CCK-8 is used to assess the cell survival of BV2 cells after treatment with ISO at various concentrations and time gradients. **(D)** BV2 cells were treated with increasing concentrations of LPS (0.5–4 μg/mL) for 24 h, followed by viability assessment using the CCK-8 assay. **(E)** BV2 cells were pretreated with different concentrations of ISO for 2 h and incubated with or without LPS (1 μg/mL) for 24 h, then cell viability was tested with the CCK-8 assay. **(F)** BV2 cells were treated with LPS (1 μg/mL) alone or in combination with ISO (8 μM) for 12 h, and their morphology was observed under an optical microscope. Scale bar: 100 µm. Results are expressed as means ± SEM, n = 3. *P < 0.05, ***P < 0.001 compared to the Control group. #P < 0.05, ###P < 0.001 when compared to the LPS group.

To determine whether ISO influences the viability of BV2 cells, the cell counting kit-8 (CCK-8) assay was performed 6 h/12 h/24 h after treatment with various concentrations of ISO ranging from 0.5 μM to 64 μM. The results (Figures 1B,C) demonstrated that ISO concentrations below 8 μM did not cause observable cytotoxic effects. Cytotoxic effects were observed above 16 μM. Consequently, concentrations of ISO within the range of 1–8 μM were utilized in subsequent experiments to ensure biological activity without compromising cell viability. To induce neuroinflammatory stress, BV2 cells were stimulated with various concentrations of LPS. LPS exposure led to abnormal proliferation of BV2 cells, reflecting the characteristic activation state induced by pro-inflammatory stimuli. Based on this (Figure 1D), a commonly used concentration of LPS was selected for subsequent experiments. Morphologically, LPS-challenged BV2 cells exhibited activated phenotypes, including enlarged cell bodies and irregular edges. The combination therapy of ISO significantly reduced these morphological changes, indicating a protective effect (Figure 1F).

### 3.2 ISO attenuates LPS-Induced neuroinflammation in BV2 cells

**FIGURE 2:**
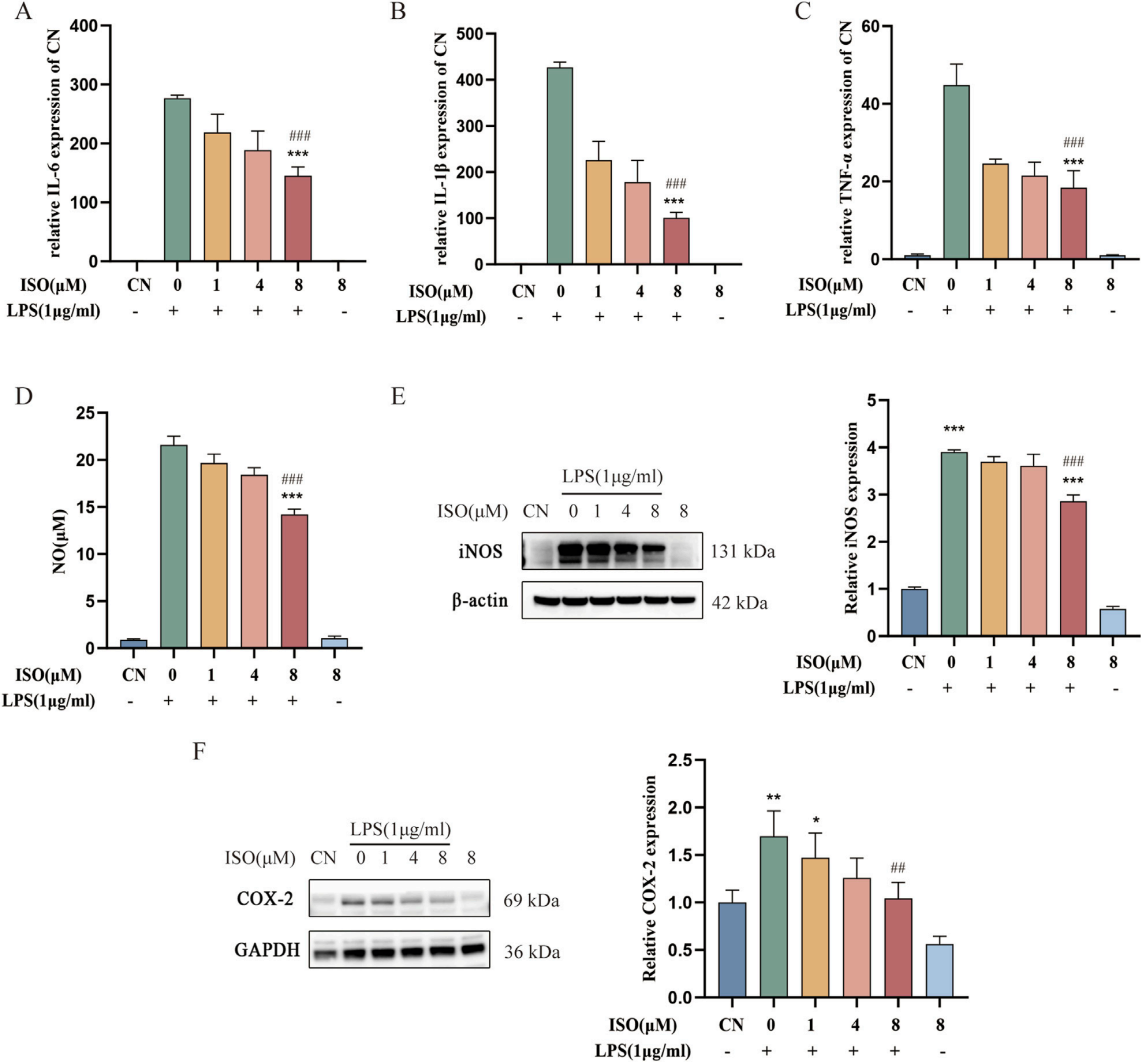
Effect of ISO on LPS-induced neuroinflammation in BV2 cells. BV2 cells were pre-treated with ISO for 2 h and then co-treated with LPS (1 μg/mL) for 6 h. The mRNA levels of IL-6 **(A)**, IL-1β **(B)**, and TNF-α **(C)** in BV2 cells treated with LPS in the presence or absence of ISO were analyzed by qRT-PCR. **(D)** BV2 cells were pre-treated with ISO for 2 h and then treated with LPS (1 μg/mL) for 24 h. The production of NO was assessed using the Griess reaction assay. **(E)** BV2 cells were pretreated with ISO for 2 h and then treated with LPS (1 μg/mL) for 12 h. The expression of iNOS and β-actin after drug treatment was measured via Western blot and quantified. **(F)** BV2 cells were pretreated with ISO for 2 h and then treated with LPS (1 μg/mL) for 12 h. The expression of COX-2 and GAPDH after drug treatment was measured via Western blot and quantified. Results are expressed as means ± SEM, n = 3. *P < 0.05, ***P < 0.001 compared to the Control group. #P < 0.05, ###P < 0.001 when compared to the LPS group.

To investigate the anti-inflammatory effects of ISO on microglial activation, BV2 cells were treated with LPS (1 μg/mL) alone or in combination with ISO at concentrations of 1, 4, or 8 μM. qRT-PCR results revealed that LPS stimulation markedly elevated the mRNA expression levels of IL-6, IL-1β, and TNF-α compared to the control group. Co-treatment with ISO significantly downregulated the expression of these pro-inflammatory cytokines, with a more pronounced effect observed at higher concentrations (Figures 2A–C). This suggests that ISO effectively mitigates the LPS-induced transcriptional activation of inflammatory mediators. In addition to its effects on cytokine transcription, ISO also influenced LPS-induced NO production. Griess assay measurements revealed that ISO co-treatment decreased the accumulation of NO in the culture medium compared with LPS stimulation alone (Figure 2D), suggesting a suppression of downstream inflammatory effector molecules. Consistently, Western blot analysis demonstrated that ISO suppressed the protein expression of inducible nitric oxide synthase (iNOS) (Figure 2E) and cyclooxygenase-2 (COX-2) (Figure 2F), both of which are key enzymes involved in inflammatory signaling pathways. These inhibitory effects were more pronounced with increasing concentrations of ISO. Collectively, these results indicate that ISO alleviates LPS-induced inflammatory responses in BV2 microglia by inhibiting the expression of pro-inflammatory cytokines and inflammation-related enzymes at both the mRNA and protein levels, thereby reinforcing the conclusion that ISO interferes with multiple components of the inflammatory cascade triggered by LPS, thereby reinforcing the conclusion that ISO interferes with multiple components of the inflammatory cascade triggered by LPS.

### 3.3 ISO suppresses LPS-induced activation of the MAPK/NF-κB pathway activation in BV2 microglial cells

**FIGURE 3:**
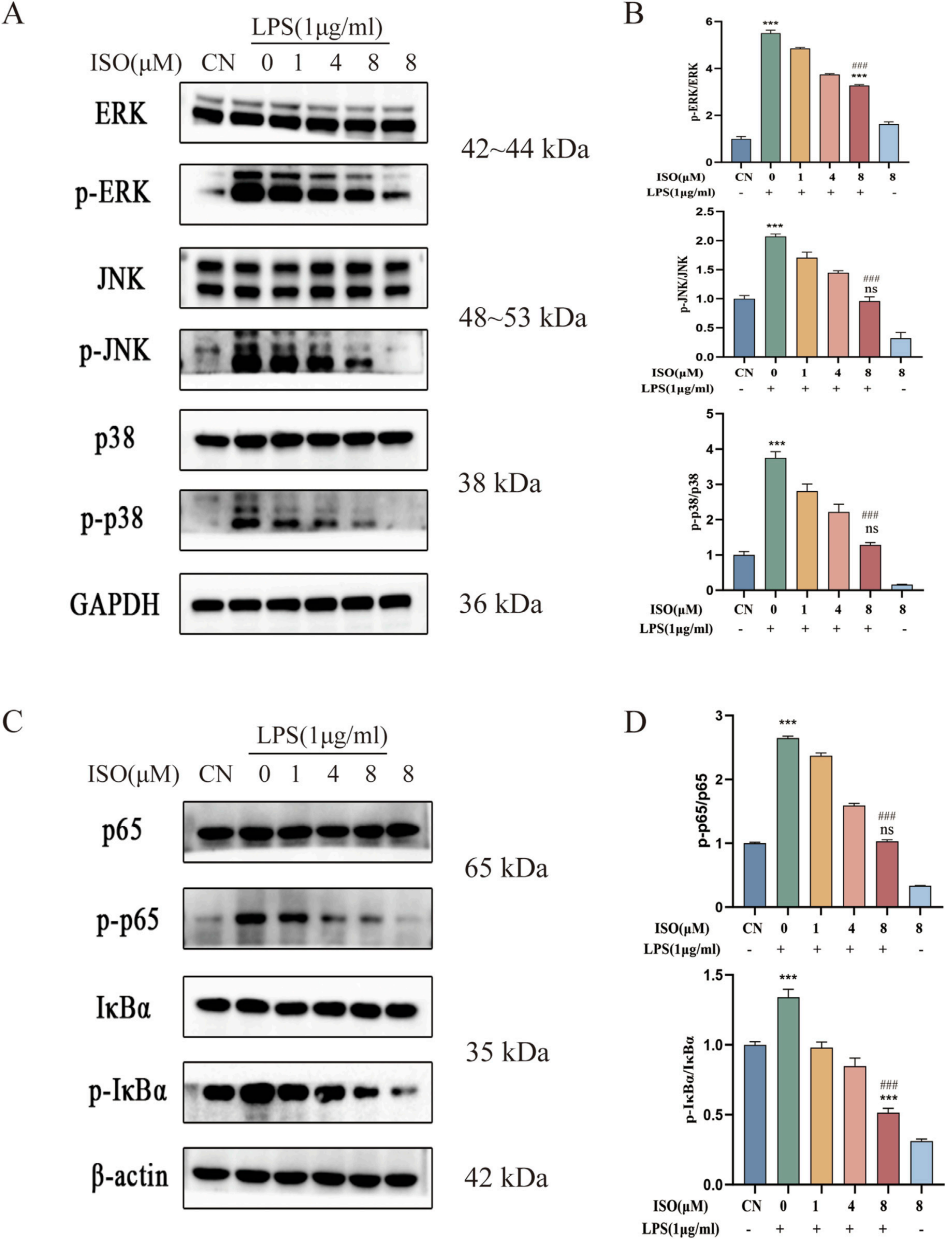
Effects of ISO on LPS-induced MAPK/NF - κB signaling pathway in BV2 cells. **(A)** BV2 cells were pretreated with ISO for 12 h, followed by LPS stimulation (1 μg/mL) for 1 h. Western blot analysis was performed to detect the expression levels of ERK, p-ERK, JNK, p-JNK, p38, and p-p38 in BV2 cells. **(B)** Quantification of relative protein expression levels in the MAPK pathway. **(C)** BV2 cells were treated under the same conditions as in **(A)**, and the expression levels of p65, p-p65, IκBα, and p-IκBα were assessed by Western blotting. **(D)** Quantification of relative protein expression levels in the NF-κB pathway. Results are expressed as means ± SEM, n = 3. *P < 0.05, ***P < 0.001 compared to the Control group. #P < 0.05, ###P < 0.001 when compared to the LPS group.

To investigate the effects of ISO on the MAPK/NF-κB signaling pathway, Western blotting was performed in BV2 cells. As expected, LPS stimulation led to a significant increase in the phosphorylation of ERK, JNK, and p38, indicating robust activation of the MAPK pathway. However, ISO treatment significantly suppressed their phosphorylation (Figures 3A,B). To further clarify the mechanistic involvement of the ERK pathway, ISO was combined with the ERK inhibitor U0126. The combined treatment produced a more pronounced inhibition of LPS-induced ERK phosphorylation compared with either treatment alone, supporting the conclusion that ISO acts, at least in part, through modulation of ERK activity (Supplementary Figure S1). Collectively, these findings demonstrate that ISO exerts anti-inflammatory effects by modulating the MAPK signaling pathway. In addition to its effects on MAPK signaling, ISO also modulated the activation of the NF-κB pathway, another central regulator of inflammatory responses. Western blot analysis demonstrated that the phosphorylation levels of IκBα and p65 were markedly increased following LPS stimulation, whereas ISO treatment significantly suppressed their phosphorylation (Figures 3C,D). To further validate the regulation of NF-κB activation, immunofluorescence staining was performed to assess p65 nuclear translocation. Consistent with the biochemical results, ISO reduced the LPS-induced accumulation of p65 in the nucleus, demonstrating that ISO interferes with the nuclear activation step required for NF-κB-mediated transcription (Supplementary Figure S2). These results indicate that ISO effectively inhibits NF-κB signaling, which is activated downstream of LPS, suggesting its potential role in regulating inflammation-related signaling cascades.

### 3.4 ISO alleviates LPS-induced oxidative stress and mitochondrial dysfunction in BV2 microglial cells

**FIGURE 4:**
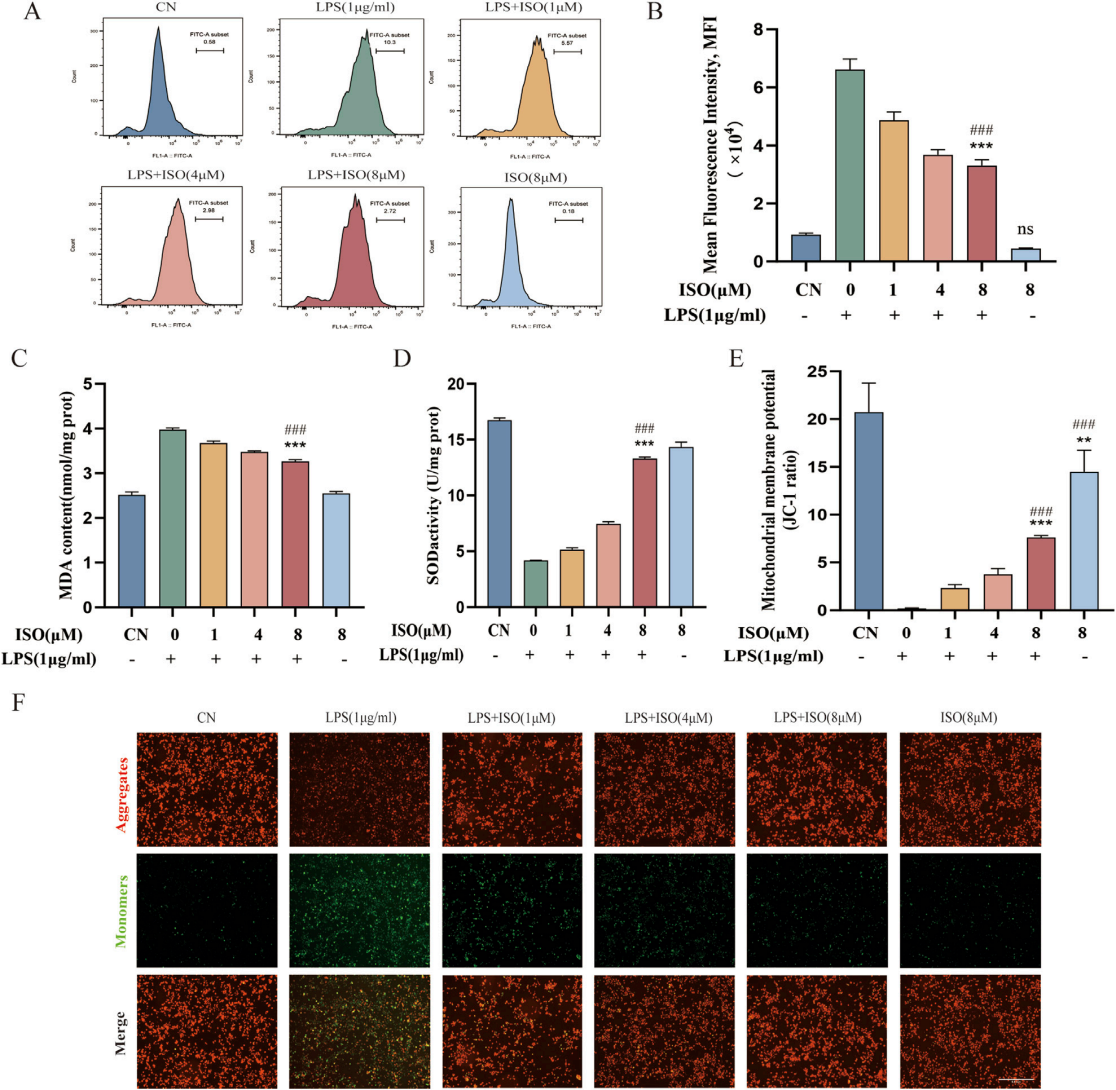
Effects of ISO on LPS-induced oxidative stress and mitochondrial dysfunction in BV2 cells. **(A)** BV2 cells were pretreated with ISO for 2 h and then treated with LPS (1 μg/mL) for 6 h. Intracellular ROS levels were detected using the DCFH-DA fluorescent probe and analyzed by flow cytometry. **(B)** Mean fluorescence intensity (MFI) quantification of ROS levels. BV2 cells were pretreated with ISO for 2 h and then treated with LPS (1 μg/mL) for 24 h. Based on the CCK-8 analysis, the levels of MDA **(C)** and SOD **(D)** were determined. **(E)** BV2 cells were pretreated with ISO for 2 h and then treated with LPS (1 μg/mL) for 6 h. Representative images from JC-1 fluorescence microscopy, red fluorescence predominantly indicates JC-1 aggregates, while green fluorescence indicates JC-1 monomers. **(F)** The red/green fluorescence ratio of JC-1 was quantified to evaluate mitochondrial membrane potential. Scale bar: 0.55 mm. Results are expressed as means ± SEM, n = 3. *P < 0.05, ***P < 0.001 compared to the Control group. #P < 0.05, ###P < 0.001 when compared to the LPS group.

To investigate the protective effect of ISO on oxidative stress in microglial cells, BV2 cells were pre-treated with ISO (1, 4, or 8 μM) for 2 h, followed by stimulation with LPS (1 μg/mL) for 6 h. Intracellular ROS levels were measured using the DCFH-DA fluorescent probe and analyzed by flow cytometry. As shown in Figure 4A, LPS stimulation significantly increased ROS production compared to the control group, while ISO pretreatment effectively reduced ROS levels. Quantitative analysis of mean fluorescence intensity (MFI) further confirmed this trend (Figure 4B). To further characterize oxidative injury, lipid peroxidation was examined by measuring MDA levels. Consistent with the observed elevation of ROS, LPS exposure led to a substantial increase in MDA content in BV2 cells, reflecting enhanced lipid oxidative damage. However, ISO treatment significantly reduced MDA accumulation (Figure 4C), indicating a protective effect against lipid oxidative damage. In addition, SOD activity, an important indicator of antioxidant defense, was found to be significantly decreased after LPS exposure. ISO reversed this reduction in SOD activity (Figure 4D), further supporting its antioxidative capacity.

To explore whether ISO also alleviates mitochondrial dysfunction, we assessed the mitochondrial membrane potential using JC-1 staining. In LPS-stimulated cells, the ΔΨm was markedly disrupted, as indicated by a shift from red to green fluorescence, suggesting depolarization of the mitochondrial membrane. ISO treatment preserved mitochondrial integrity, as evidenced by increased red/green fluorescence ratio (Figures 4E,F). This preservation of ΔΨm indicates that ISO protects mitochondria from LPS-induced depolarization and dysfunction. Collectively, these results demonstrate that ISO effectively mitigated oxidative stress and maintained mitochondrial homeostasis in activated microglia. By reducing excessive ROS accumulation and preventing mitochondrial depolarization, ISO may help sustain cellular redox balance and energy metabolism, thereby limiting inflammation-related cellular injury.

### 3.5 ISO protects HT-22 cells against LPS-Induced neurotoxicity indirectly

**FIGURE 5:**
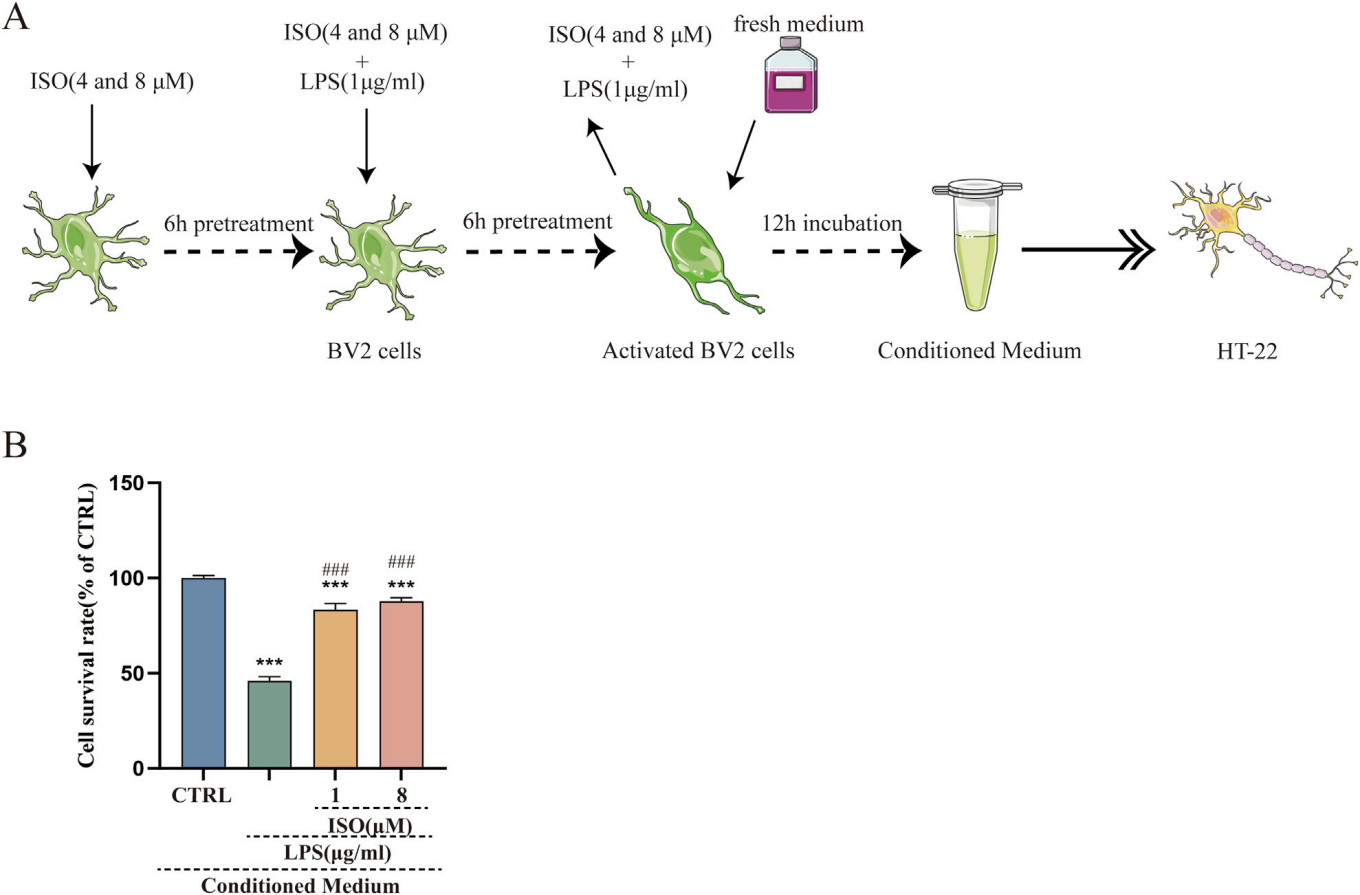
Neuroprotective effects of ISO on HT-22 cells cultured with conditioned medium from LPS-stimulated BV2 cells. **(A)** A diagram of the toxic effect of conditioned media from LPS-stimulated BV2 cells on HT-22 cells with or without the ISO pretreatment. **(B)** HT-22 cells were treated with conditioned medium for 24 h, and cell viability was assessed using CCK-8 assays. Results were shown as means ± SEM, *P < 0.05, **P < 0.01 and ***P < 0.001 vs. HT-22 cells exposed in conditional medium from CTRL BV2 cells, ##P < 0.01, ###P < 0.001vs. HT-22 cells exposed in conditional medium from LPS-stimulated BV2 cells.

Microglia were capable of detecting extracellular alterations. Upon LPS stimulation, these cells released a variety of cytokines, chemokines, and other soluble mediators into the surrounding microenvironment, ultimately influencing neuronal activity and viability. The neuron-microglia co-culture system effectively modeled microglia-mediated neurotoxicity and was routinely employed to investigate neuroinflammatory mechanisms in AD pathogenesis (Jian et al., 2019). Direct co-culture might introduce artifacts due to variable cell ratios and contact modes, whereas conditioned medium (CM) allowed isolated analysis of microglial secretome effects (Gao et al., 2023). In the present, our data confirmed that treatment with ISO significantly suppressed the production of pro-inflammatory cytokines and NO in LPS-stimulated BV2 microglial cells, demonstrating its ability to suppress microglial inflammatory activation. To further determine whether this anti-inflammatory shift translated into neuroprotective benefits, HT-22 neuronal cells were exposed to CM collected from treated BV2 cells. HT-22 cells treated with CM derived from LPS-stimulated BV2 cells exhibited a significant reduction in cell viability compared to the control group (P < 0.01, Figure 5B). However, pretreatment with 2 or 8 μM ISO-CM markedly ameliorated this viability reduction. These results demonstrate that ISO pretreatment effectively reversed microglia-induced neurotoxicity, providing protection for HT-22 cells against pro-inflammatory insults.

## 4 Discussion

Neuroinflammation is a critical mechanism underlying the initiation and progression of neurodegenerative diseases, particularly AD. Initially, as the intrinsic immune cells of the central nervous system, microglia play a pivotal role in maintaining brain homeostasis and conducting immune surveillance (Wang et al., 2023). In the early stages of AD, microglia recognize and attempt to clear pathological Aβ plaques, while also secreting neuroprotective substances such as nerve growth factor (NGF) to support neuronal survival and tissue integrity (Baligacs et al., 2024). However, as the disease progresses, these microglia undergo polarization, transitioning to a pro-inflammatory phenotype (M1 type) (AmeliMojarad and AmeliMojarad, 2024). M1-polarized microglia release high levels of pro-inflammatory cytokines, including TNF-α and IL-1β, along with excessive ROS (Guo et al., 2022). These inflammatory mediators not only directly injure surrounding neurons and synaptic structures but also facilitate further Aβ deposition and promote hyperphosphorylation of tau, thereby contributing to the formation and exacerbation of neurofibrillary tangles (Zhang et al., 2024a). Moreover, dysfunctional microglia exhibit impaired capacity to clear Aβ plaques, resulting in progressive accumulation of Aβ within the brain. This process establishes a detrimental feedback loop involving persistent inflammation, Aβ deposition, and neuronal injury, which markedly accelerates AD-associated neurodegeneration (Ma et al., 2023). Consequently, neuroinflammation has a multifaceted negative impact on the pathological development of AD (Zhang et al., 2023). Therefore, targeting microglia-driven molecular pathways represents a promising strategy to address specific aspects of neuroinflammation and provide reliable therapeutic interventions to halt or slow the progression of AD.

Currently available pharmacological treatments for AD includes cholinesterase inhibitors, NMDA receptor antagonists, and Aβ-targeting agents under clinical investigation. Nonetheless, none of these therapies offer a curative effect (Tolar et al., 2020). Specifically, cholinesterase inhibitors (e.g., donepezil, rivastigmine, and galantamine) ameliorate cognitive function by inhibiting acetylcholinesterase activity, thereby elevating acetylcholine levels in the brain (He and Tam, 2024). Similarly, Memantine hydrochloride, an NMDA receptor antagonist, exerts neuroprotective effects by mitigating glutamate-induced excitotoxicity (He and Tam, 2024). Nevertheless, these conventional AD therapies provide only symptomatic relief without modifying disease progression, failing to address the multifactorial pathogenesis of AD. As a result, single-target drugs are inadequate for modulating such complex pathological cascades. In contrast, natural compounds exhibit multi-target and multi-pathway potential due to their structural diversity and bioactivity, offering novel therapeutic avenues for AD (Bhat et al., 2022). For instance, curcumin has been demonstrated to reduce amyloid plaque formation, suppress tau phosphorylation, and modulate neuroinflammation via pathways such as NF-κB and MAPK (Serafini et al., 2017). Meanwhile, resveratrol activates SIRT1 to enhance Aβ clearance while conferring neuronal protection via antioxidant mechanisms (Hao et al., 2023). In a similar vein, ISO, as a natural compound, likely derives its therapeutic potential from the concurrent modulation of multiple pathways. We acknowledge that its specificity may not be absolute, yet this very characteristic may enable it to exert broader neuroprotective effects through synergistic actions on multiple targets. Previous studies have demonstrated that ISO can protect HT-22 neuronal cells against glutamate-induced mitochondrial membrane potential loss and intracellular iron overload while also inhibiting the accumulation of MDA and ROS in both cytosolic and lipid compartments.

Therefore, to evaluate the potential neuroprotective effects of ISO, this present study elucidated the beneficial effects of ISO on LPS-stimulated BV2 cells and HT-22 cells cultured with conditioned medium. Our findings demonstrate that ISO exerts anti-inflammatory effects by modulating the MAPK/NF-κB signaling pathway, thereby reducing a cascade of inflammatory responses. Furthermore, ISO alleviates oxidative stress and mitochondrial dysfunction—two key pathological features of AD—which collectively contribute to its neuroprotective properties. These results highlight the unique advantages of ISO as a multi-target compound capable of modulating multiple aspects of AD pathogenesis, warranting further investigation in primary microglia and *in vivo* models to explore its underlying neuroprotective mechanisms in greater depth.

In patients with AD, pro-inflammatory cytokines such as IL-1β, IL-6, and TNF-α are significantly elevated in brain tissue, cerebrospinal fluid (CSF), and serum. These cytokines are primarily secreted by microglia and astrocytes that have been persistently activated by β-amyloid deposition, thereby contributing to neurotoxicity, disruption of the blood–brain barrier (BBB), and the hyperphosphorylation of tau protein (Janelidze et al., 2018). Moreover, in AD, microglia undergo a phenotypic shift toward the pro-inflammatory M1 state, characterized by the upregulation of iNOS, which catalyzes the production of NO (Guo et al., 2022). Notably, excessive NO is neurotoxic, as it can react with superoxide anions to form peroxynitrite (ONOO^−^), thereby exacerbating neuronal damage (Si et al., 2023). Additionally, COX-2 is another key pro-inflammatory enzyme involved in the biosynthesis of inflammatory mediators such as prostaglandin E2 (PGE2) (Kumari et al., 2024). Therefore, assessing the protein expression levels of iNOS and COX-2 provides valuable insight into the activation status of inflammation-associated enzymes.

Furthermore, activated microglia tend to aggregate around Aβ plaques, forming an “inflammatory encapsulation” that further undermines the BBB and facilitates the entry of harmful substances into the CNS, thus perpetuating a vicious cycle of inflammation (Eimer et al., 2018). In this study, pretreatment with ISO significantly reduced LPS-induced upregulation of iNOS and COX-2 protein expression, as well as the production of NO in BV2 microglial cells. Moreover, ISO treatment markedly attenuated the LPS-induced elevation of IL-6, IL-1β, and TNF-α mRNA levels (see Figure 2). These findings indicate that ISO exerts neuroprotective effects, at least in part, by suppressing M1-type polarization of microglia and thereby mitigating neuroinflammation. However, the present study primarily focused on the pro-inflammatory M1 phenotype, and the potential effects of ISO on the anti-inflammatory M2 phenotype were not assessed. Therefore, future investigations should explore whether ISO can promote the shift of microglia from the M1 to the M2 phenotype, which would provide valuable insights into the comprehensive neuroprotective mechanisms of ISO. Notably, HT-22 neuronal cells cultured with conditioned medium from ISO-pretreated BV2 cells exhibited significantly enhanced viability. This effect is likely due to the reduced release of pro-inflammatory cytokines and NO from activated BV2 cells, highlighting the potential of ISO as an anti-inflammatory agent (see Figure 5). However, a key limitation of the current research is the lack of direct quantification of inflammatory cytokines secreted in conditioned media, which requires ELISA detection of specific soluble mediators (such as IL-1 β, IL-6 and TNF-α) to confirm the mechanistic pathways. This will also be a priority in future research.

The observed anti-inflammatory effects of ISO suggest that its mechanism of action may not be limited to the direct suppression of inflammatory factors, but rather is likely achieved by interfering with upstream signal transduction events. Given that the initiation and amplification of inflammatory responses are highly dependent on key intracellular signaling pathways, particularly the MAPK and NF-κB pathways, which are recognized as master switches regulating the expression of inflammatory factors. Although its role via the MAPK/NF-κB pathway has been reported in osteoarthritis models (Zhang et al., 2024b), the immune privilege of the central nervous system and the unique response mechanisms of microglia imply potential differences in the signal transduction network. Therefore, directly confirming the regulatory effect of ISO on the MAPK/NF-κB pathway within the specific pathophysiological context of neuroinflammation is an indispensable step for elucidating its central anti-inflammatory mechanism. To further investigate the downstream molecular mechanism underlying the anti-inflammatory effects of ISO, we next employed Western blot analysis to detect its impact on the phosphorylation levels of key proteins in the MAPK and NF-κB signaling pathways. LPS, a constituent of the outer membrane of Gram-negative bacteria, functions as a ligand for TLR4, initiating downstream signaling cascades. Following TLR4 activation, the myeloid differentiation primary response protein 88 (MyD88)-dependent pathway initiates the activation of the IκB kinase (IKK) complex. The IKK complex promotes the phosphorylation and subsequent degradation of IκBα, thereby releasing NF-κB dimers to translocate into the nucleus and bind to specific DNA sequences to initiate the transcription of pro-inflammatory cytokines (Zheng et al., 2020). Simultaneously, activation of MAPK kinases leads to the phosphorylation and activation of several transcription factors, such as AP-1, therefore modulating the expression of inflammation-related genes (Wang et al., 2024). As expected, Western blot analysis revealed that LPS stimulation significantly increased the phosphorylation of ERK, JNK, and p38 (see Figure 3A), along with marked phosphorylation of IκBα and nuclear translocation of phosphorylated NF-κB (see Figure 3B), in comparison to the control group. Notably, ISO treatment attenuated the LPS-induced activation of the NF-κB and MAPK pathways and inhibited p65 nuclear translocation. Co-treatment with the ERK inhibitor U0126 further suppressed ERK phosphorylation, thereby exerting anti-inflammatory effects (see Supplementary Figures S1, S2). Meanwhile, further exploration is needed in the future to use MAPK inhibitors or activators to simulate or reverse the effects of ISO on pro-inflammatory markers.

Inflammation frequently coincides with oxidative stress. MDA, a key lipid peroxidation byproduct, can be detected during the early stages of AD progression. Immunoelectron microscopy has revealed MDA deposits in glial cells encircling lipid droplets in AD patients (Dei et al., 2002). Notably, elevated MDA levels have been reported to correlate positively with the severity of neuronal damage and show a significant association with cognitive impairment (Tang et al., 2022). The human brain demands substantial energy to sustain proper neuronal activity and cognitive functions. In AD patients, individual mitochondria frequently exhibit swelling and cristae disruption (Pradeepkiran and Reddy, 2020), leading to reduced ATP levels and abnormal MMP in neurons (Ashleigh et al., 2023). This dysfunction compromises ATP synthesis efficiency and results in excessive ROS production, which overwhelms the cellular antioxidant defense system and further exacerbates oxidative stress (Pradeepkiran et al., 2022). To evaluate the functional effects of ISO, we employed DCFH-DA and JC-1 fluorescent probes to assess intracellular ROS accumulation and MMP in BV2 microglial cells. Additionally, levels of MDA and SOD were analyzed. The results demonstrated that ISO treatment alleviated intracellular oxidative stress in a dose-dependent manner, contributed to the normalization of MMP, and reduced mitochondrial damage (see Figure 4). In summary, our findings highlight the therapeutic potential of ISO in mitigating oxidative stress and attenuating mitochondrial dysfunction.

In this study, we selected the murine BV2 microglial cell line as our *in vitro* model. This choice was based on its practical advantages, including ease of culture, genetic homogeneity, and consistent response to inflammatory stimuli, which provide a robust and reproducible platform for mechanistic investigation (Kong et al., 2022). We acknowledge the inherent limitations of the BV2 model. As an immortalized cell line, BV2 cells may not fully recapitulate the gene expression profiles and functional complexity of primary or *in vivo* microglia, particularly in terms of inflammatory sensitivity and cytokine secretion spectra. Despite these limitations, the LPS-induced BV2 model remains a valuable and well-established tool in neuroinflammatory research. It effectively mimics the core processes of microglial activation (Xie et al., 2025). LPS reliably triggers microglial activation through TLR4-mediated signaling, resembling the innate immune response observed in the early phase of AD before extensive Aβ deposition. Moreover, this model provides high reproducibility and clear inflammatory responses, making it suitable for evaluating anti-inflammatory interventions. Utilizing this model, we aimed to precisely delineate the regulatory effects of ISO on microglial inflammatory responses. Our findings provide crucial *in vitro* evidence for understanding its potential neuroprotective mechanisms and form a solid foundation for subsequent *in vivo* studies. Although the present study focused on microglia–neuron interactions, it remains to be determined whether ISO directly affects MAPK signaling or inflammatory responses in neuronal cells such as HT-22, which will be addressed in future work.

To the best of our knowledge, this study is the first to demonstrate that ISO exerts a straightforward neuroprotective effect in a LPS-induced neuroinflammatory model through both anti-inflammatory and antioxidant properties. Moreover, this study provides the first evidence that ISO modulates the MAPK/NF-κB signaling pathways in this cellular context, offering a clearer mechanistic insight into how ISO confers neuroprotection. In particular, the suppression of pro-inflammatory cytokines and the protection against oxidative stress and mitochondrial dysfunction are of critical importance, as these processes are closely associated with the pathogenesis of AD. More importantly, our findings support the MAPK/NF-κB signaling cascade as a potential therapeutic target for neuroinflammatory conditions. Although this study confirms the beneficial effects of ISO on neuroinflammation and its modulation of the MAPK/NF-κB pathway, it should be noted that, given the multifactorial nature of AD pathology, our investigation represents only an initial step. Further studies are warranted to explore the underlying mechanisms in greater depth. Since our study was primarily conducted in a cellular model, the neuroprotective effects and associated molecular mechanisms of ISO have yet to be validated *in vivo*. Therefore, investigating the protective effects of ISO and elucidating its molecular mechanisms in animal models of AD will be valuable avenues for future research.

## 5 Conclusion

**FIGURE 6:**
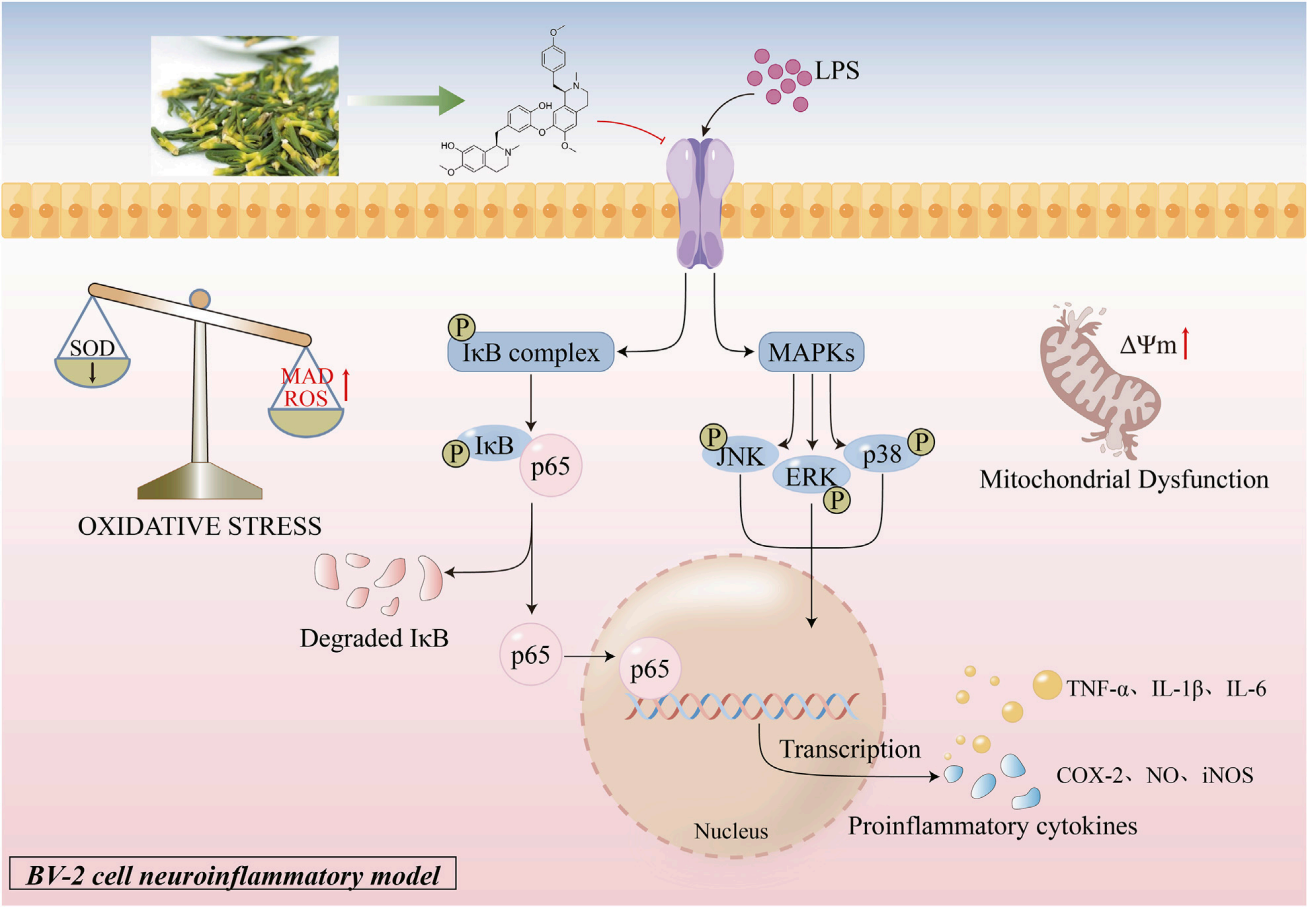
ISO reduces neuroinflammation via MAPK/NF-κB signaling modulation and alleviates oxidative stress and mitochondrial damage, contributing to neuroprotection.

In this study, we systematically demonstrated that ISO attenuates LPS-induced neuroinflammation in BV2 microglial cells by suppressing MAPK/NF-κB signaling. It also diminishes oxidative stress and safeguards mitochondrial activity, collectively enhancing its neuroprotective properties (Figure 6). To our knowledge, this study is the inaugural demonstration that ISO provides neuroprotection by concurrently modulating inflammation, oxidative stress, and mitochondrial integrity. These findings suggest that ISO may has therapeutic potential in decelerating the progression of neurodegenerative diseases such as AD.

## Data Availability

The original contributions presented in the study are included in the article/Supplementary Material, further inquiries can be directed to the corresponding author.
