# Supplementary material for: Isoliensinine confers neuroprotection and alleviates LPS-induced neuroinflammation in microglia by regulating the MAPK/NF-κB signaling
Source: Front Pharmacol. 2025 Dec 10;16:1675865. doi: 10.3389/fphar.2025.1675865 (PMC12727965; doi:10.3389/fphar.2025.1675865)
Supplement: Supplementary file 1 [file Supplementaryfile1.docx]

Supplementary Material

# Original western blots description: All protein bands are repeated three times (marked as red 1, 2, 3 in the picture). Each protein band contains the grayscale, brightfield, and merge images of the protein and the corresponding internal reference (They are marked as PUB, Maker, PUB+Maker in the figure).

## Original western blots for Figure 2E (iNOS, β-actin)


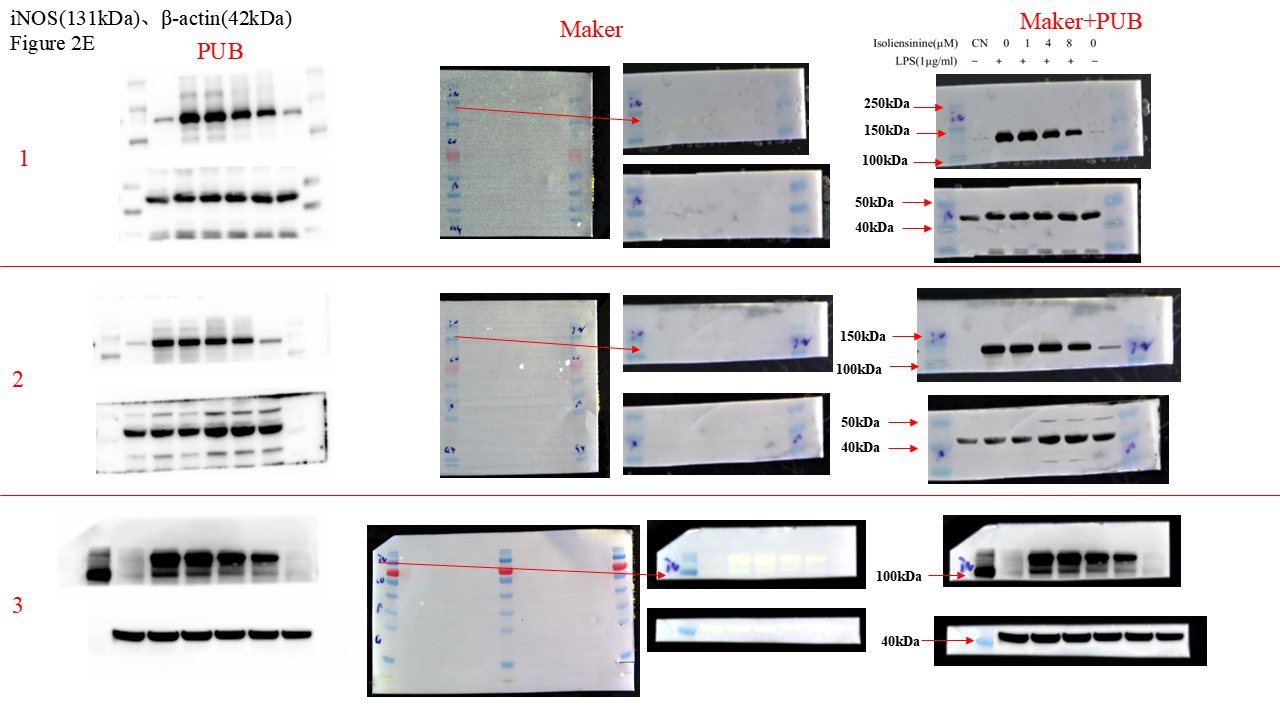


## Original western blots for Figure 2E (COX-2, GAPDH)


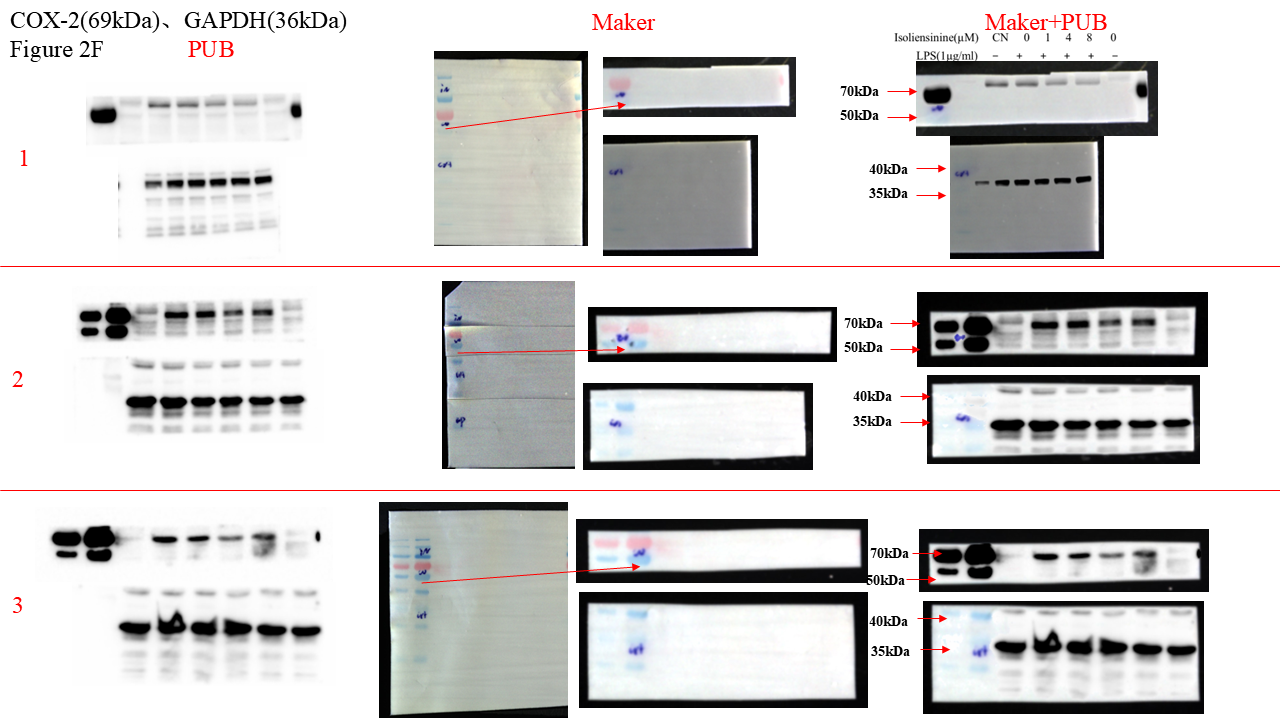


## Original western blots for Figure 3A(ERK, p-ERK, GAPDH)


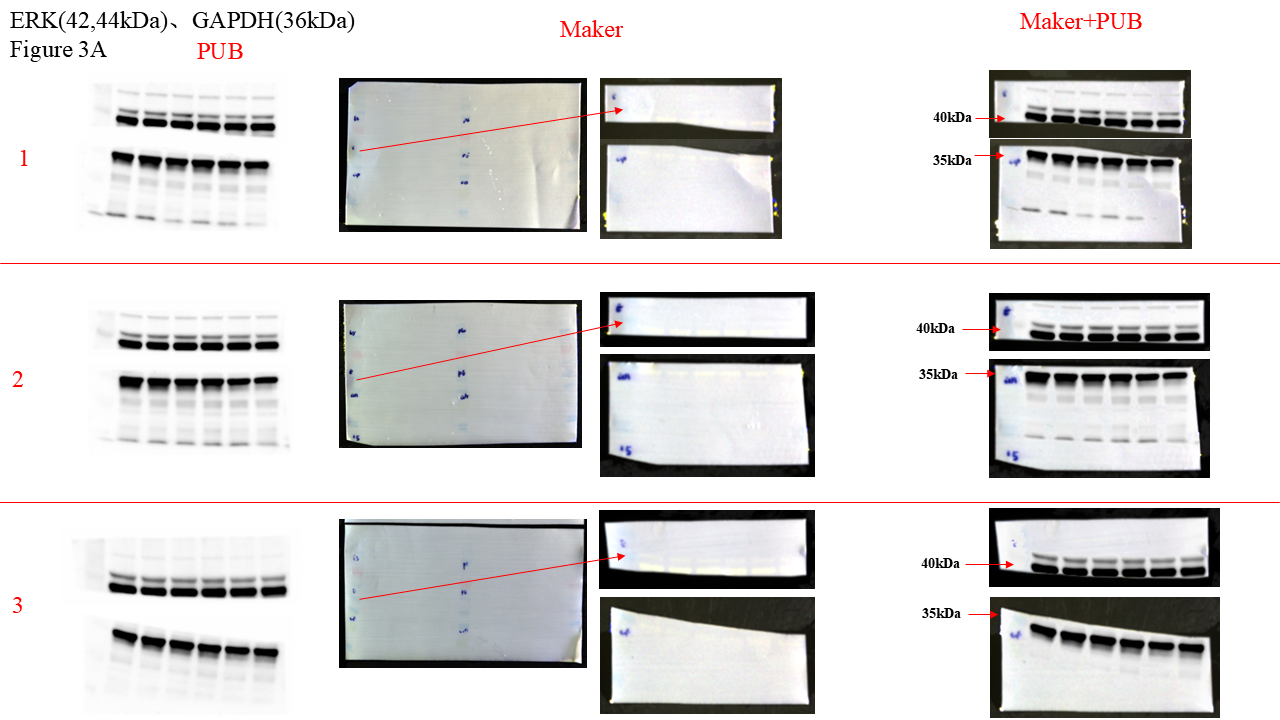


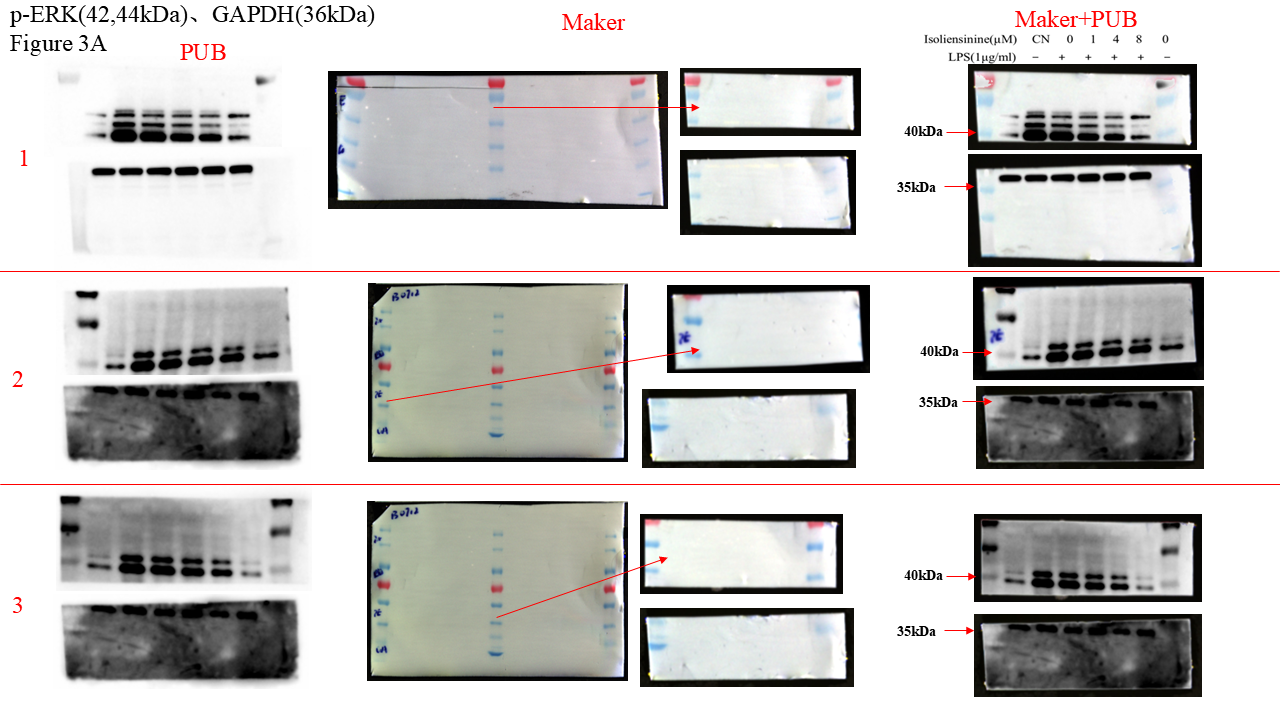


## Original western blots for Figure 3A (JNK, p-JNK, GAPDH)


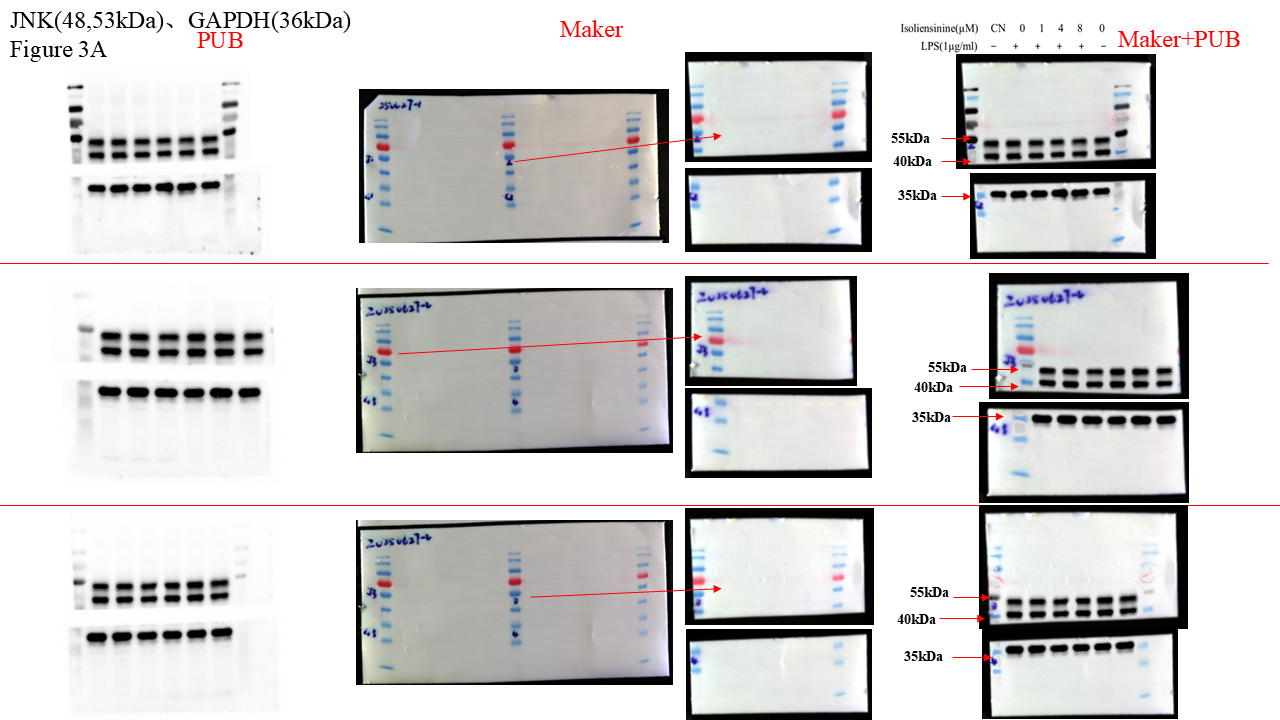


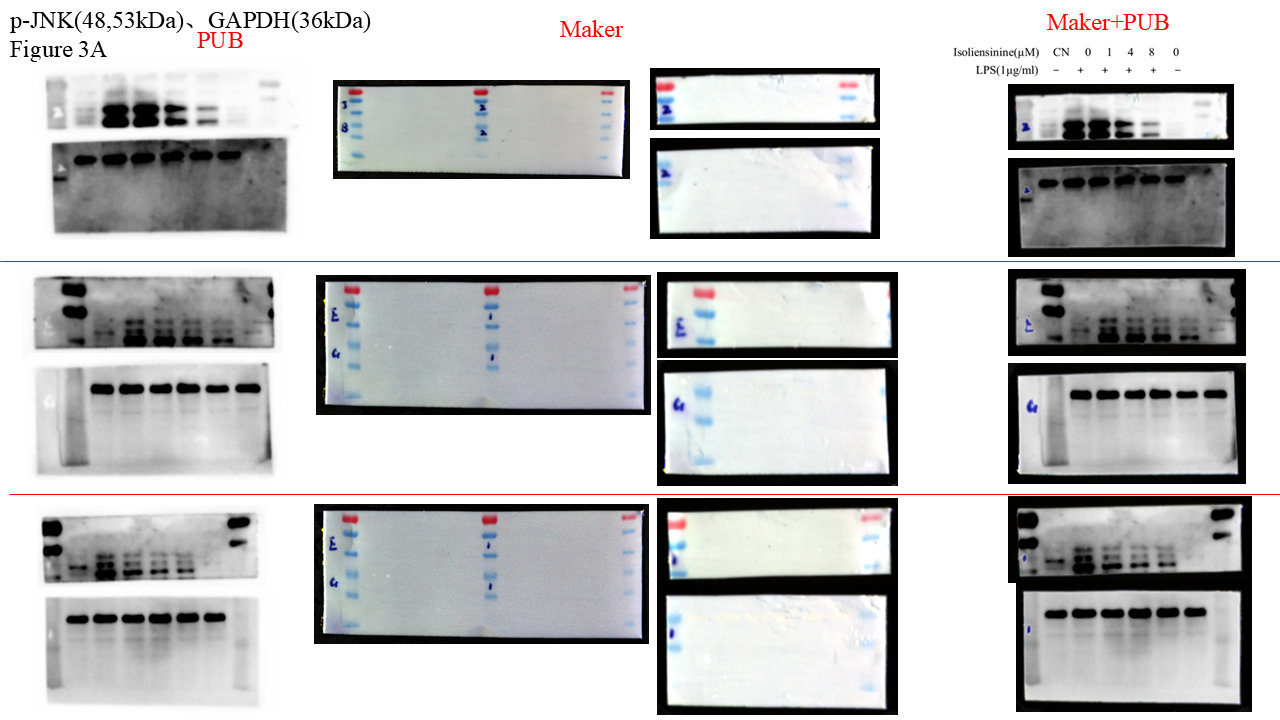


## Original western blots for Figure 3A (p38, p-p38, GAPDH)


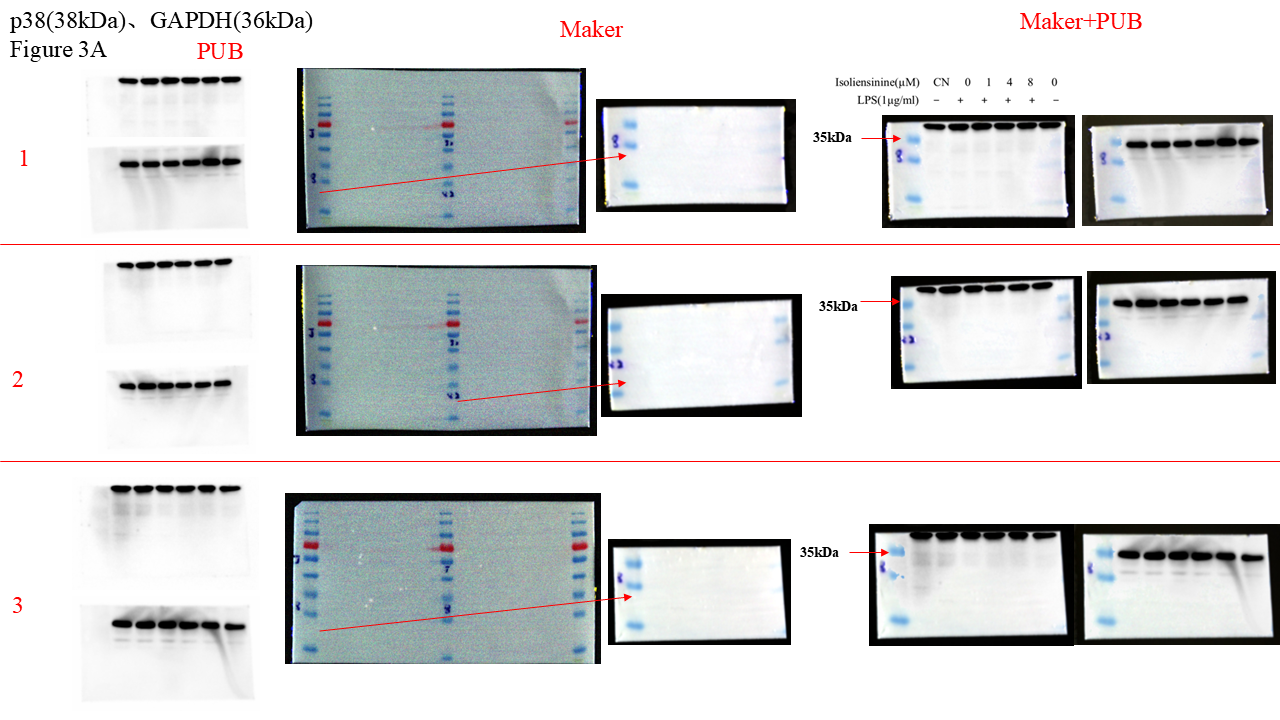


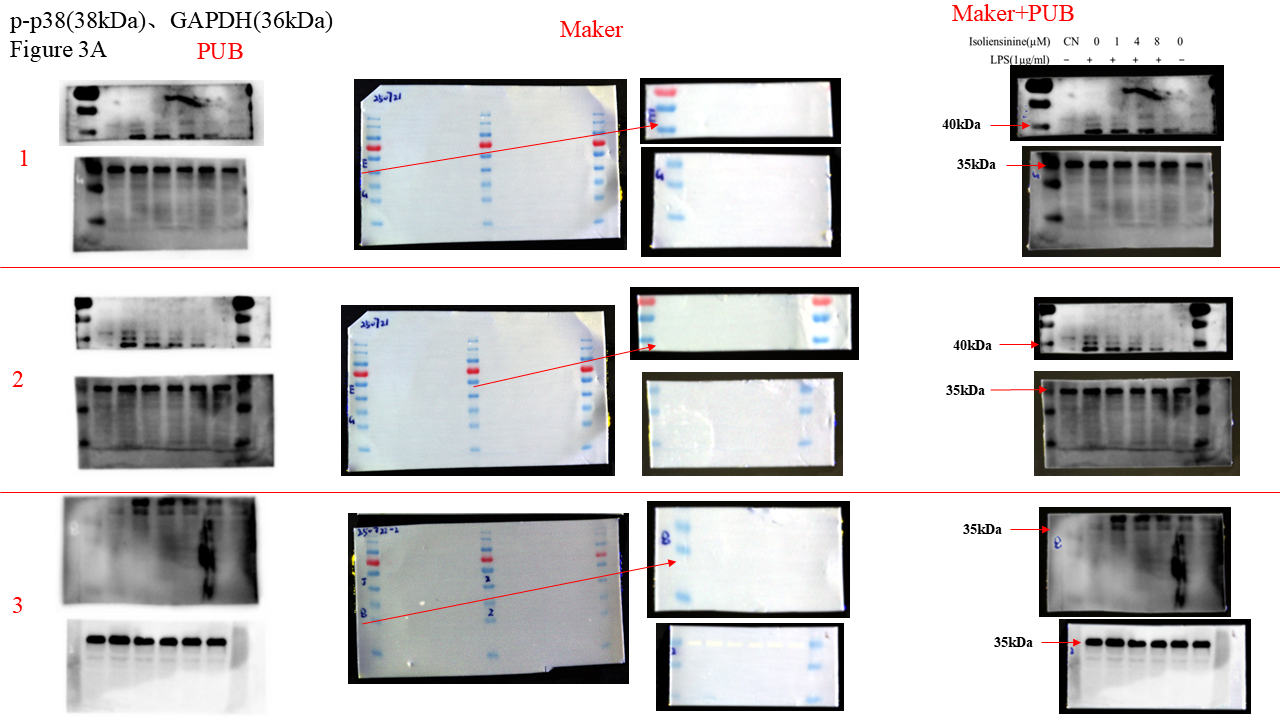


## Original western blots for Figure 3B (p65, p-p65, β-actin)


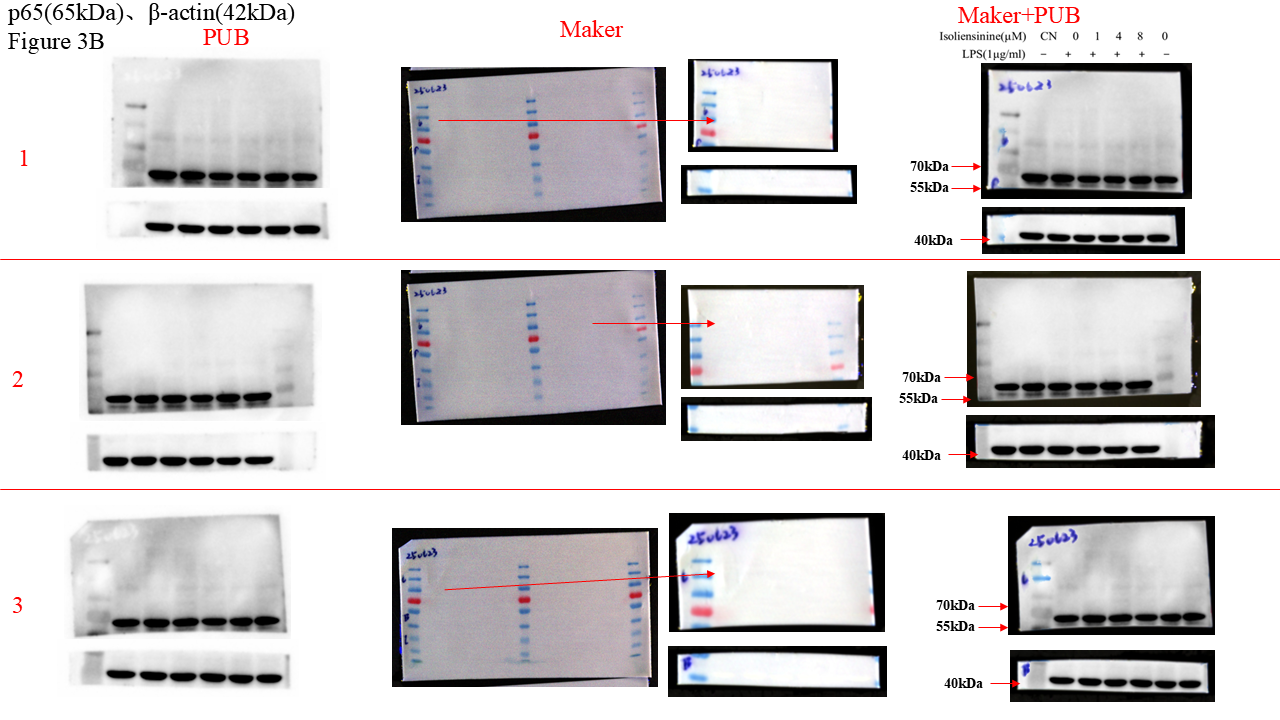


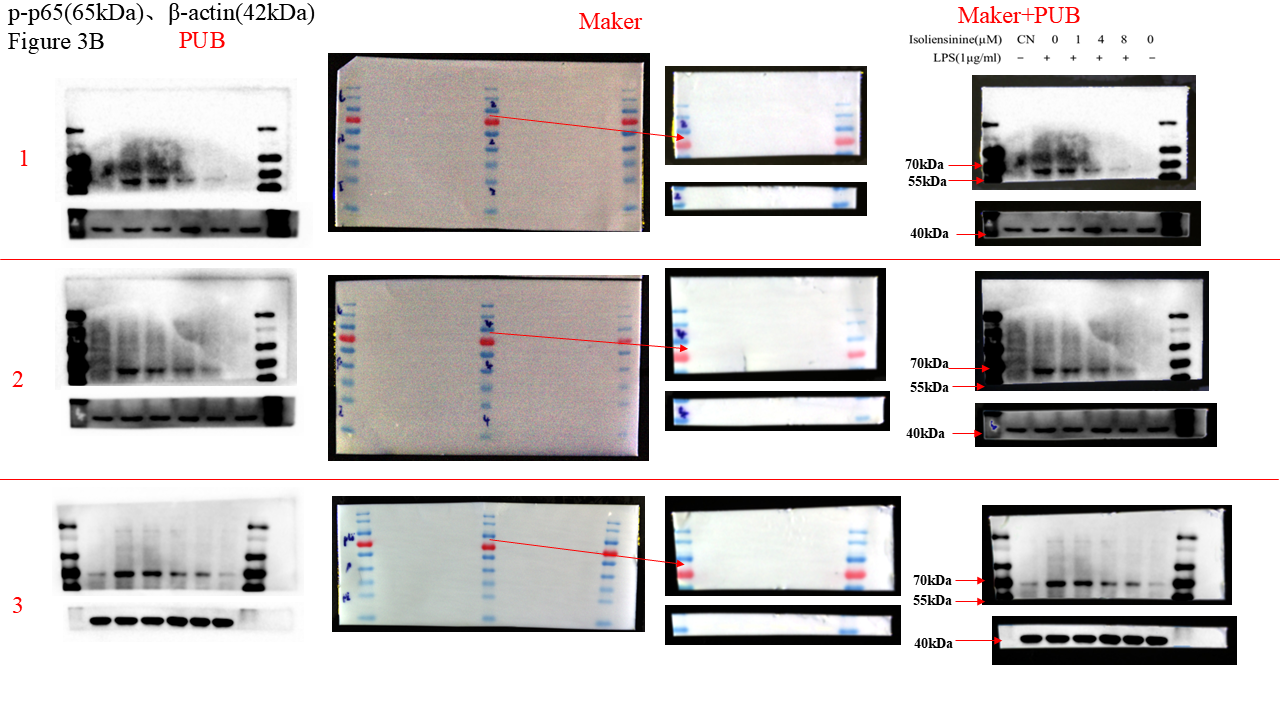


## Original western blots for Figure 3B (IκBα, p- IκBα, β-actin)


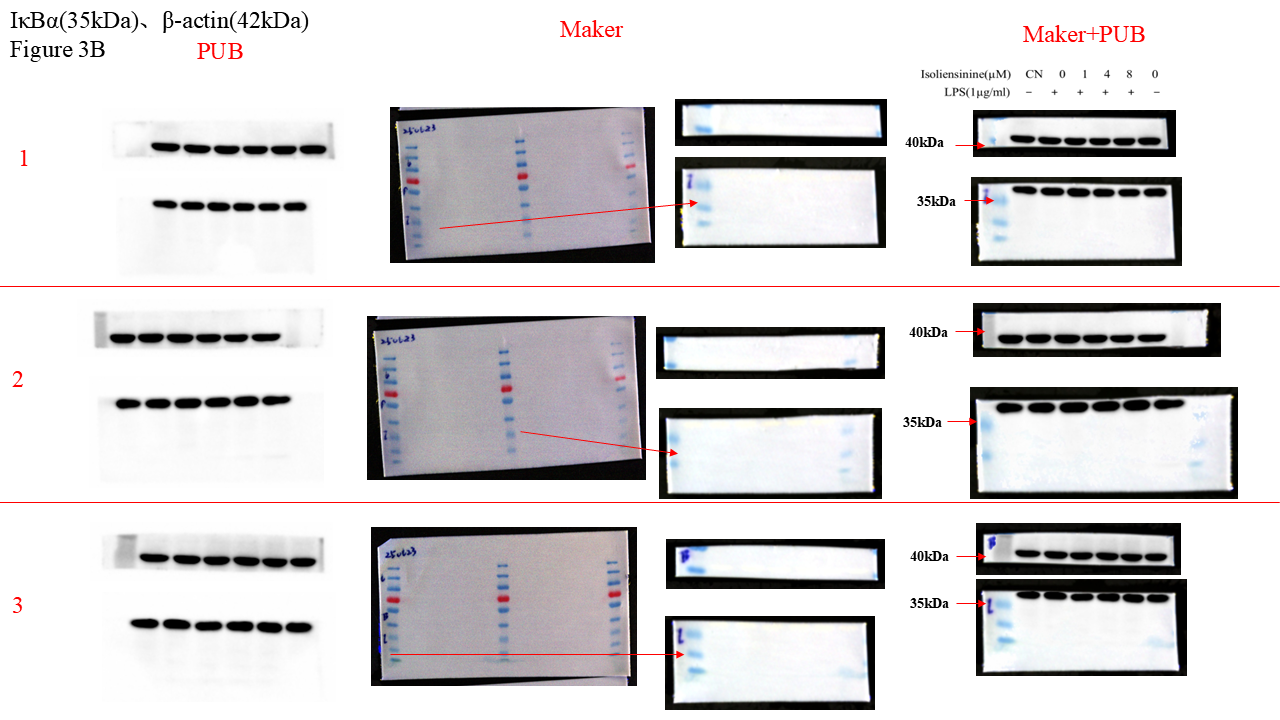


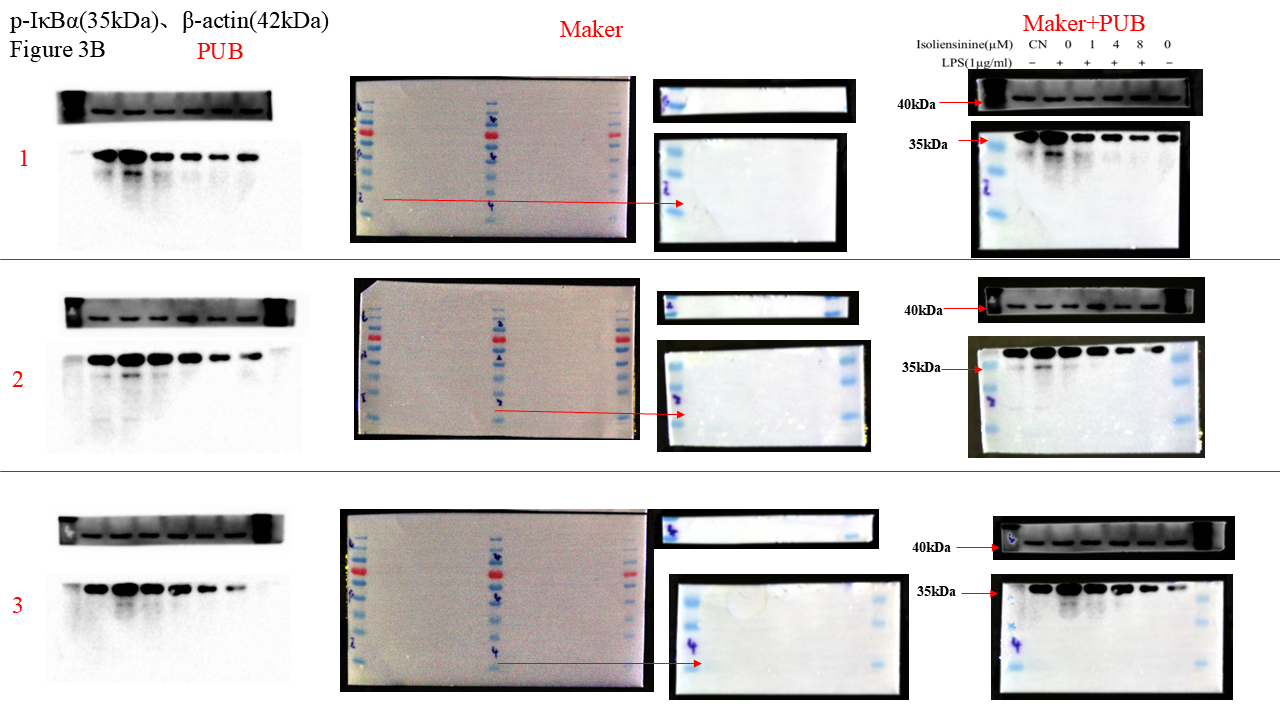


## Original western blots for Supplement Figure 1A (ERK, p-ERK, GAPDH)


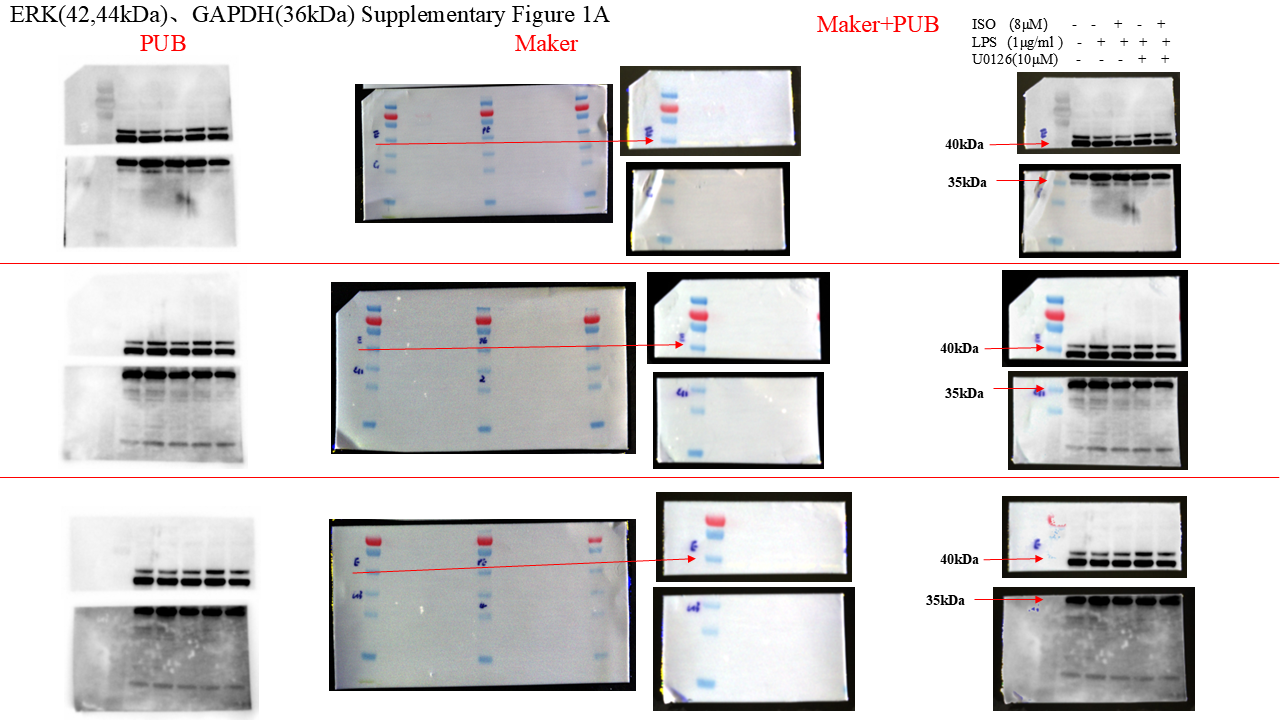


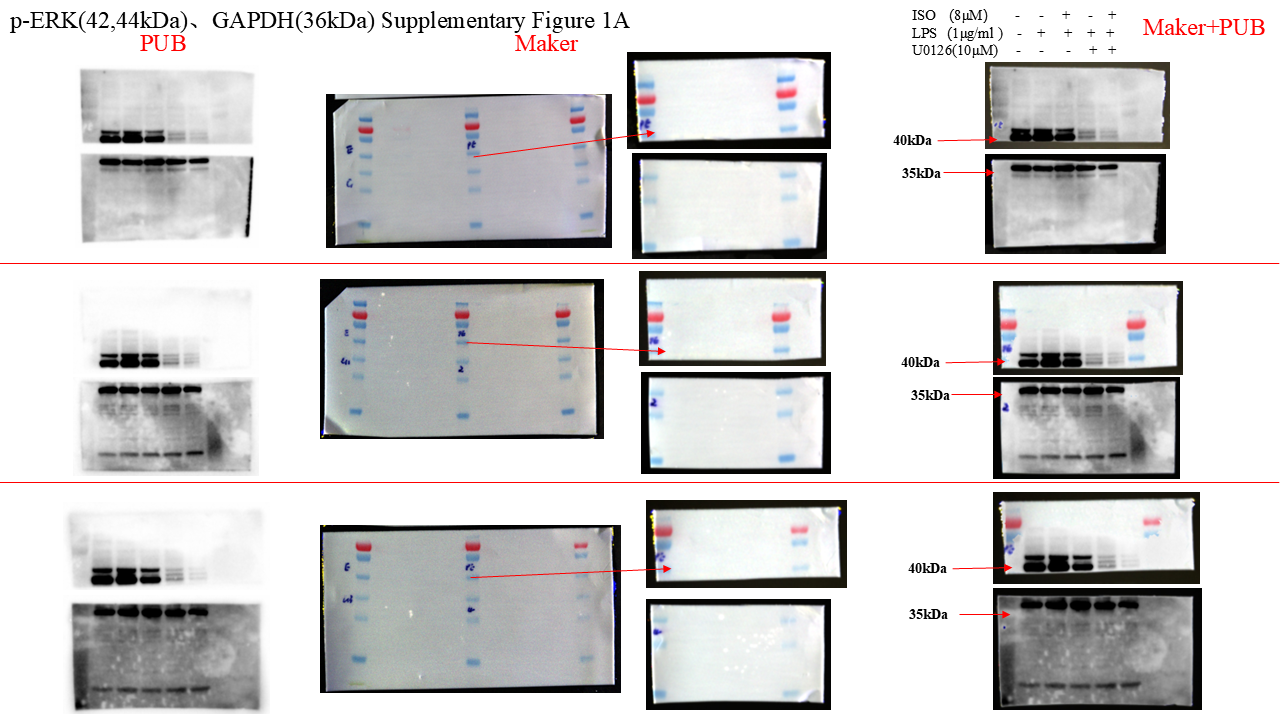


# Supplementary Figures


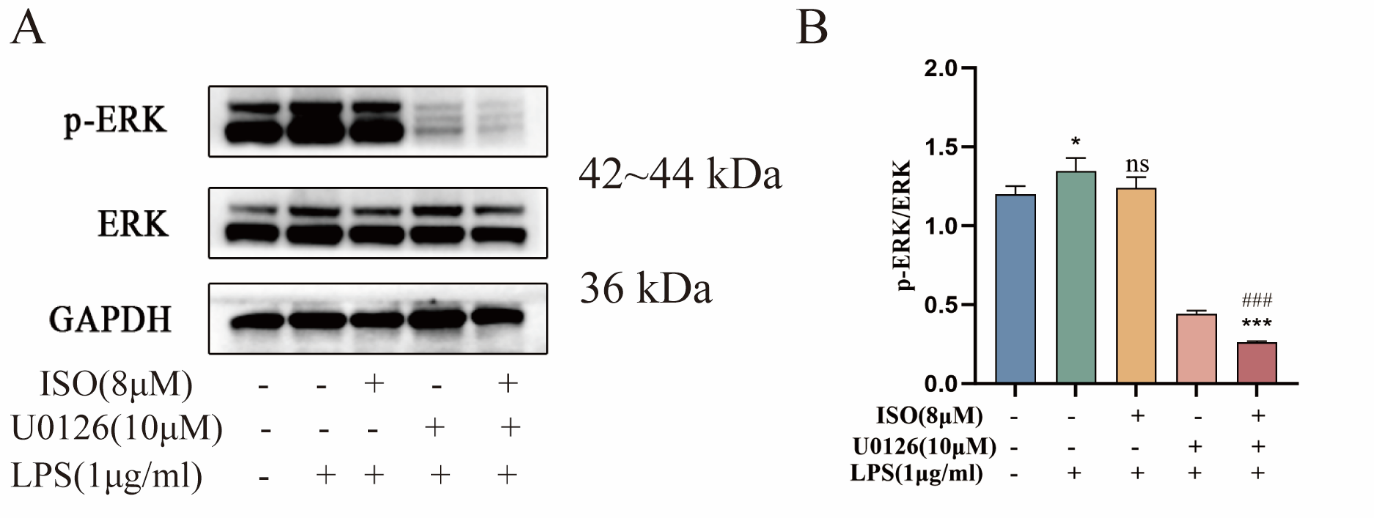


Supplementary Figure 1.

Effect of ISO and U0126 on LPS-induced ERK phosphorylation in BV2 microglial cells. (A) BV2 cells were pre-treated with ISO (8 μM) or/and U0126 (10 μM) for 12 h and then co-treated with LPS (1 μg/mL) for 1 h. The protein levels of p-ERK and total ERK were determined by Western blot analysis. (B) Quantitative analysis of the p-ERK/ERK ratio. GAPDH or β-actin was used as a loading control to confirm consistent protein loading across all samples. Results are expressed as means ± SEM, n = 3. * P < 0.05, *** P < 0.001 compared to the Control group. # P < 0.05, ### P < 0.001 when compared to the LPS group.


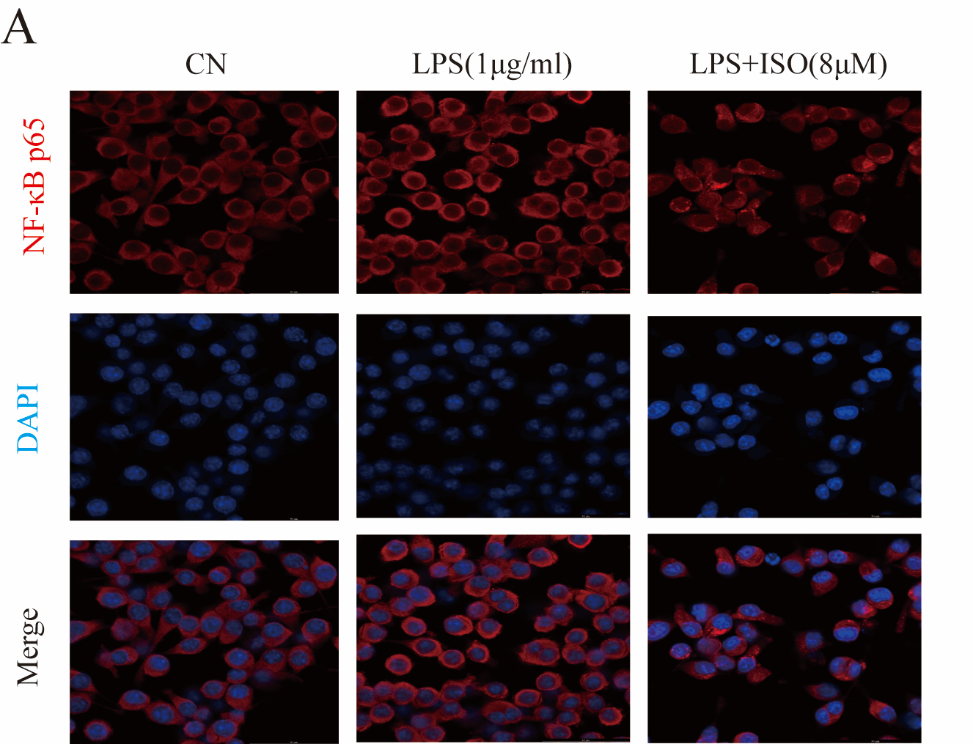


Supplementary Figure 2.

BV2 cells were pre-treated with ISO for 12 h and then co-treated with 5 (1 μg/mL) for 6 h. The translocation of NF-κBp65 (red) to the nucleus (blue) is shown by confocal microscopic images. Scale bar: 50 µm.


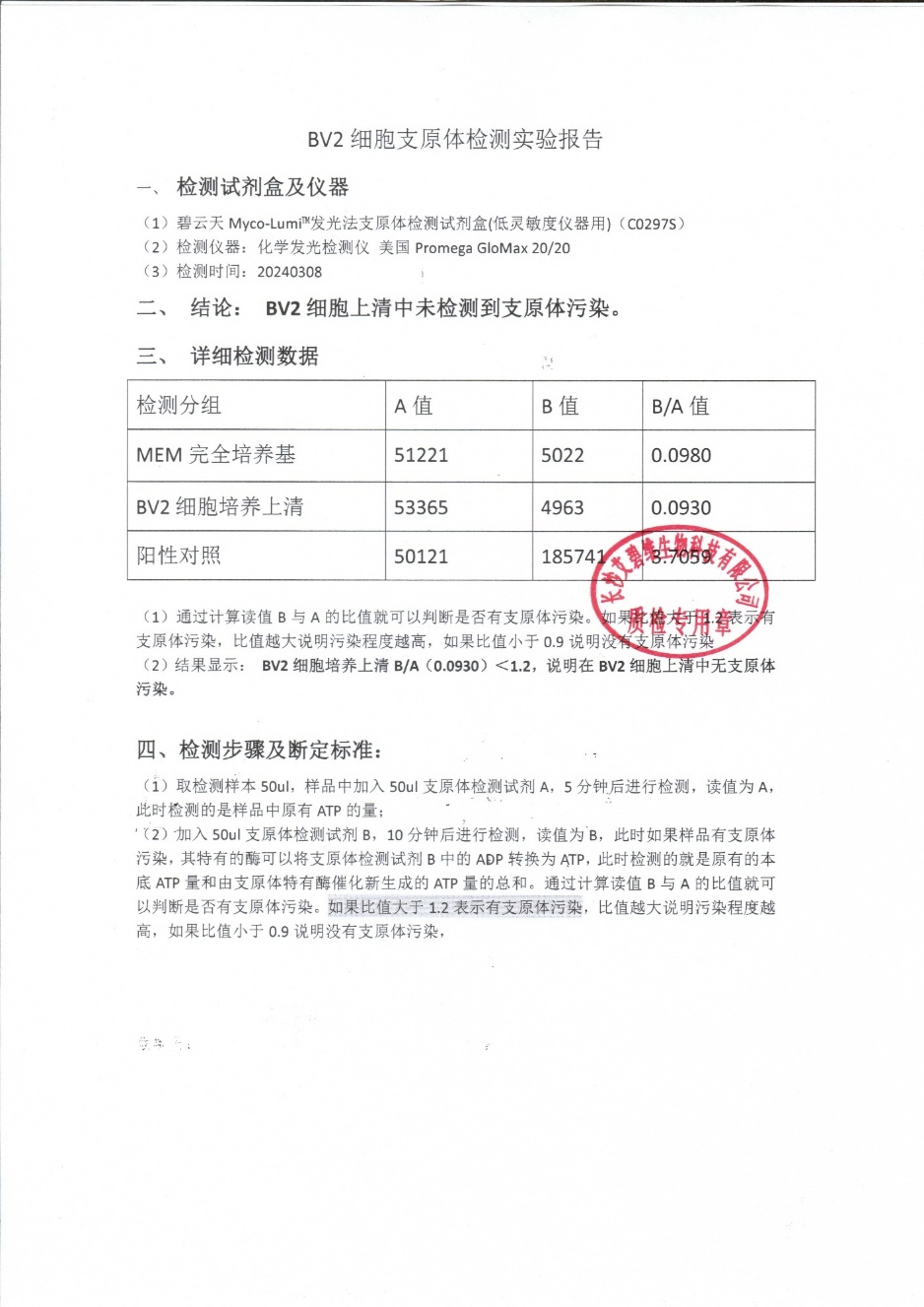


Supplementary Figure 3.

Mycoplasma testing report. As shown in Supplementary Figure 3, in the fourth column of the third point's table, the B/A ratio is used to measure whether the cells are contaminated by mycoplasma. This result indicates that the B/A ratio of the BV2 cell supernatant is 0.930, which is similar to that of the complete MEM medium and much lower than the critical value of 0.9 for mycoplasma contamination. This suggests that the BV2 cells we cultivated were not contaminated by mycoplasma.The testing was provided by Abiowell Biotechnology Co., Ltd. (Changsha, China).


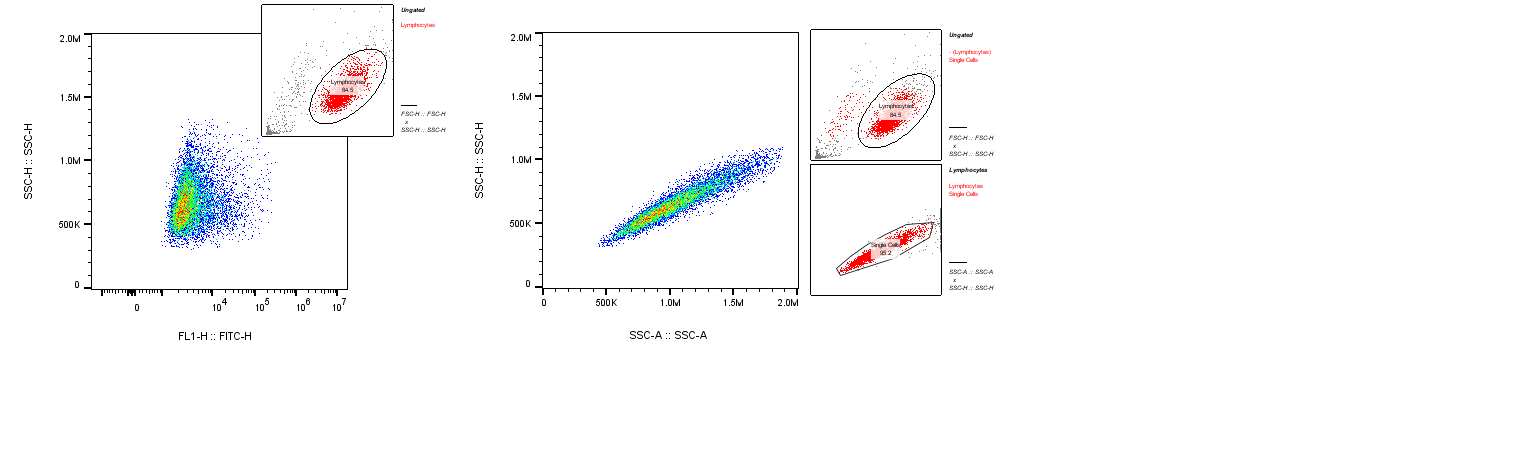


Supplementary Figure 4. Gating strategy for flow cytometry analysis.

Representative plots illustrating the gating procedure used to identify the analyzed cell populations. (Left) Cells were first gated on SSC-A versus FSC-A to exclude debris and select the main cell population. (Right) Single cells were further identified based on SSC-H versus SSC-A to remove doublets. Fluorescence intensity in the FITC channel (FL1-H) was then analyzed to determine ROS-positive cells. Insets show the defined gates for lymphocyte-like populations and corresponding single-cell distributions.
